# NOTCH3 Mutation Causes Glymphatic Impairment and Promotes Brain Senescence in CADASIL

**DOI:** 10.1111/cns.70140

**Published:** 2025-01-24

**Authors:** Chunyi Li, Hui Li, Xuejiao Men, Yuge Wang, Xinmei Kang, Mengyan Hu, Xiaotao Su, Shisi Wang, Danli Lu, Shishi Shen, Huipeng Huang, Xiaohui Deng, Yuxin Liu, Lei Zhang, Wei Cai, Aimin Wu, Zhengqi Lu

**Affiliations:** ^1^ Department of Neurology, Mental and Neurological Disease Research Center The Third Affiliated Hospital of Sun Yat‐Sen University Guangzhou China; ^2^ Department of Cerebrovascular Disease The Fifth Affiliated Hospital of Sun Yat‐Sen University Zhuhai China; ^3^ Guangdong Provincial Key Laboratory of Brain Function and Disease Guangzhou China

**Keywords:** AQP4, brain senescence, CADASIL, glymphatic system

## Abstract

**Aims:**

The aim of this study is to investigate the role of glymphatic function of cerebral autosomal dominant arteriopathy, subcortical infarcts, and leukoencephalopathy (CADASIL), the most common monogenic small vessel disease caused by *NOTCH3* mutation, and to explore potential therapeutic strategies to improve glymphatic function.

**Methods:**

We assessed glymphatic influx and efflux function in CADASIL mouse models (*Notch3* ^R170C^) and correlated these findings with brain atrophy in CADASIL patients. We also investigated the underlying mechanisms of glymphatic impairment, focusing the expression of AQP4 in astrocytic endfeet.

**Results:**

CADASIL mouse exhibited both impaired glymphatic influx and efflux, which impedes waste clearance and promotes brain senescence. In accordance, brain atrophy in CADASIL patients is associated with perivascular space enlargement, indicating that glymphatic impairment contributes to advanced brain senescence in CADASIL. The glymphatic malfunction in CADASIL is attributed to diminished AQP4 expression in astrocytic endfeet, which is the core mediator of glymphatic activity. Mechanistically, AQP4 expression is regulated by NOTCH3‐RUNX1‐CMYB signaling. Reinforcing AQP4 expression in astrocytes by AAV‐based therapy resumes the glymphatic functions in CADASIL mice, which further prevents brain senescence.

**Conclusion:**

We propose that to improve glymphatic function by reinforcing AQP4 expression is a promising therapeutic strategy in CADASIL.

## Graphical abstract

Beside mural cells, NOTCH3 is expressed in astrocytes. Expression of Aquaporin 4 (AQP4), the core mediator of glymphatic functions, is decreased in CADASIL brain. Consequently, glymphatic activities are impaired. Diminished AQP4 expression is attributed to the suppression of RUNX1‐CMYB‐AQP4 signaling due to NOTCH3 mutation in astrocytes. To reinforce AQP4 expression by astrocyte‐targeting AAV resumes glymphatic functions in CADASIL mice, which subsequently prevents brain senescence.

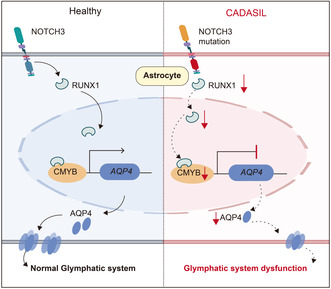

**Abbreviations**
AAV: adeno‐associated virus
AQP4: aquaporin 4
AUC: area under curve
BBB: blood–brain barrier
BN page: blue native page
CADASIL: cerebral autosomal dominant arteriopathy, subcortical infarcts, and leukoencephalopathy
CM: cisterna magna
CNS: central nervous system
CSF: cerebrospinal fluid
DAC: dystrophin‐associated complex
EB: evans blue
EPVS: enlargement of perivascular space
GCA: global cortex atrophy
GOM: granule osmiophilic material
ISF: interstitial fluid
LDH: lactate dehydrogenase
MRI: magnetic resonance imaging
OAPs: orthogonal arrays of particles
OSCs: organotypic slice cultures
PVS: perivascular space
ROC: receiver operating characteristic
SA‐β‐GAL: senescence‐associated β galactosidase
SPP: signaling pathways project
TEM: Transmission electron microscopy
TFs: transcription factors

## 1 Introduction

Cerebral autosomal dominant arteriopathy, subcortical infarcts, and leukoencephalopathy (CADASIL) is the most common monogenic small vessel disease which is caused by mutation of *NOTCH3* gene. Patients of CADASIL usually meet disease onset in their prime years with initial symptoms of migraine or psychiatric disorders. As the disease progresses, patients come across recurrent lacunar infarction and white matter degeneration and mostly culminate in vascular dementia [1]. The underlying mechanisms of CADASIL pathophysiology remain largely elusive. As a result, specific treatment against CADASIL is scarce up to date [2]. *Runx1* is a NOTCH target gene and NOTCH activation upregulates RUNX1 expression [3, 4, 5]. RUNX1 then translocates into the nucleus and mediates gene transcription [6, 7] with various cofactors including CMYB [8, 9, 10]. NOTCH‐RUNX1‐CMYB pathway exerts is implicated in vital physiologic processes such as stem cell development [4, 11]. Nevertheless, functional alterations of NOTCH downstream pathways in CADASIL, including the RUNX1‐CMYB signaling, remain to be studied.

It has been widely documented that patients with CADASIL display brain volume decrement [12, 13]. According to previous report, brain atrophy is one of the most sensitive neuroimaging features of CADASIL [14]. Besides, senile plaque, which is a feature of brain aging and degenerative diseases, is also found in the brain lesions of CADASIL patients [15], indicating that CADASIL brains might undergo advanced senescence.

Glymphatic system, a network of perivascular tunnels wrapped by astrocyte endfeet, is responsible for waste removal from the central nervous system (CNS). Aquaporin 4 (AQP4), the water channel expressed on the endfeet of astrocytes, enable rapid interstitial fluid (ISF) transport by glymphatic system [16]. Proper function of glymphatic system is critical for brain homeostasis maintenance, while dysfunction of glymphatic system exacerbates neurofunctional deficits [17]. Glymphatic malfunction could cause enlargement of perivascular space (EPVS) as revealed by neuroimaging [18]. Accumulative evidence reveal that increased EPVS burden is commonly manifested in CADASIL patients [19, 20]. Furthermore, NOTCH3 is predominantly expressed in vascular smooth muscle cells and pericytes, while absence of *Notch3* leads to glymphatic dysfunction and neurodegeneration [21]. Since CADASIL is a genetic disorder linked to *NOTCH3* missense mutations, this suggests the implication of glymphatic impairment in CADASIL progression.

Glymphatic functions decline with age [22, 23, 24], which results in delayed removal of metabolic waste and accumulation of harmful substances. Consequently, glymphatic dysfunction during aging further promotes brain senescence [22]. Considering the detrimental impacts of glymphatic failure to brain senility, we hypothesize that glymphatic system is impaired in CADASIL, which exacerbates brain senescence.

The current study explores functional alteration of glymphatic system and the consequent impacts to brain senescence. We report that brain atrophy in CADASIL patients is associated with the deteriorated EPVS burden. With the CADASIL mouse model of *Notch3* ^R170C+/+^ (NOTCH3‐R170C), we find that both the cerebrospinal fluid (CSF)‐to‐ISF influx and ISF‐to‐periphery efflux are retarded in CADASIL. The hypofunction of glymphatic system is attributed to the low AQP4 expression in astrocytes due to reduced activation of NOTCH3‐RUNX1‐CMYB signaling. Brain senescence is evident as early as 16 weeks of age in CADASIL mice. Reinforcing AQP4 expression in astrocytes significantly enhances glymphatic function and prevents brain senescence. We thus propose that to improve glymphatic function is a promising therapeutic strategy against brain presenility in CADASIL.

## 2 Material and Methods

### 2.1 Study Population

A cohort consisted 27 patients with CADASIL in neurology clinics and 29 age‐ and sex‐matched healthy controls (HC) in medical examination department in the Third Affiliated Hospital of Sun Yat‐sen University from July 2019 to December 2023 was recruited consecutively. Inclusion process is shown in Figure S1 and cohort demographics is displayed in Table S1. Standard MRI were performed in all recruited patients for neuroimaging assessment. Diagnoses of all CADASIL patients recruited in the study were confirmed by genetic testing in all cases, which is the gold standard for the diagnosis of CADASIL [1]. The healthy controls had no neurologic or psychiatric diseases or disease‐associated harbor mutations in the coding sequence of *NOTCH3*, and their MRI did not meet the imaging criteria for cerebral small vessel disease [25]. Portraits of PVS in MRI is fluid‐filled space following the course of penetrating vessel of cerebrospinal fluid signal on all sequences, which can be either linear (when parallel to the penetrating vessel), round or ovoid (when perpendicular to the penetrating vessel) with a diameter < 3 mm. In biologic context, PVS is hardly detected. However, enlarged PVS (EPVS) could be observed. Grading scale of EPVS is as follows: Grade 0 indicates no EPVS, Grade 1 indicates 1–10 EPVS, Grade 2 indicates 11–20 EPVS, Grade 3 indicates 21–40 EPVS, and Grade 4 indicates the presence of more than 40 EPVS [26]. Brain atrophy, which is not related to macroscopic injuries including trauma or stroke, was defined according to GCA (Global cortex atrophy) rating. The GCA scale assesses the extent of atrophy in the cortex and sulcal dilatation, with scores ranging from 0 to 3. The scoring system is as follows: Grade 0 indicates no cortical atrophy; Grade 1 indicates mild atrophy with some opening of sulci; Grade 2 indicates moderate atrophy with significant volume loss of gyri; and Grade 3 indicates severe atrophy characterized by “knife blade atrophy” [27].

### 2.2 MRI Protocol and Neuroimaging Assessment

MRI was performed on a GE 3.0‐Tesla scanner MR750 (General Electric, Milwaukee, USA) with a standard eight‐channel HRBRAIN coil. The MRI protocol included (i) axial T1 FLAIR (fluid‐attenuated inversion recovery) weighted: repetition time (TR) = 1750 ms, echo time (TE) = 24 ms, echo train length (ETL) = 10, bandwidth (BW) = 41.67 kHz, matrix = 320 × 224, filed of view (FOV) = 240 mm, slice thickness = 5 mm, spacing = 1, and number of excitations (NEX) = 1; (ii) axial T2‐weighted FrFSE (fast recovery fast spin echo): TR = 5727 ms, TE = 93 ms, ETL = 32, BW = 83.3 kHz, matrix = 512 × 512, FOV = 240 mm, slice thickness = 5 mm, spacing = 1, and NEX = 1.5; (iii) T2 FLAIR weighted: TR = 8400 ms, TE = 145 ms, inversion time (TI) = 2100 ms, BW = 83.3 kHz, flip angle (FA) = 145°, matrix = 320 × 224, FOV = 240 mm, slice thickness = 5 mm, spacing = 1, and NEX = 1; (iv) axial three‐dimensional time‐of‐flight MR angiography (3D‐TOF MRA): TR = 25 ms, TE = 3.4 ms, FA = 20°, BW = 41.67 kHz, matrix size = 384 × 320, FOV = 200 mm, slice thickness = 0.8 mm, and NEX = 1; (v) Axial T2*‐weighted angiography (SWAN): TR = 77.3 ms, TE = 45 ms, BW = 62.5 kHz, FA = 15°, matrix = 384 × 320, slice thickness = 1 mm, and NEX = 1. MRI DICOM (Digital Imaging and Communications in Medicine) data were analyzed by an experienced neuroradiologist (X.C.) in ORS Visual (Montreal, Quebec, Canada). Image processing was performed with MATLAB using SPM12 package (SPM12, 2014) and the CAT12 toolbox. Briefly, a preprocessing pipeline within the CAT12 toolbox, based on SPM12, was employed for the following sequential analyses: functional realignment, motion estimation, structural segmentation and normalization, normalization of functional images to the Montreal Neurological Institute (MNI) template, detection of functional outliers, application of a band‐pass filter ranging from 0.01 to 0.1 Hz, and regression of confounding factors (white matter, cerebrospinal fluid, realignment parameters, and artifact scrubbing). The processed T1‐weighted images yielded data from 170 brain regions as delineated by the Automated Anatomical Labeling (AAL) atlas 3. Subsequently, the total gray matter volume and the volumes of distinct brain lobes were computed.

### 2.3 Animals

Wild‐type (WT, C57/BL6), NOTCH3‐R170C mice were purchased from Shanghai Model Organisms Center Inc. and were bred in the South China Agricultural University. The construction of NOTCH3‐R170C mice has been described previously [28]. Female and male mice were group‐housed a 12:12 light/dark cycle, temperature with 24°C ± 2°C, humidity between 30% and 70%, with ad libitum access to food and water. Male and female mice were used with a distribution of 45%–55% of each gender in the groups.

### 2.4 Drug Treatment

C57/BL6 mice (16w of age) anesthetized with 1.25% tribromoethanol at 50 g/mL and the CM were exposed. A 30‐gauge needle filled with the TGN‐020 (MCE, HY‐W008574) or PBS was inserted into the CM. Five microliters (5 mg/mL) of TGN‐020 or PBS was infused at a rate of 2.5 μL min − 1 for 2 min. Needle were withdrawn after 5 min and scalps were cleaned and sutured with sterile surgical sutures. The TGN‐020 or PBS was administered twice a week for 1 week.

### 2.5 Transmission Electron Microscopy (TEM)

Fresh brain tissues were collected using a sharp blade within 1–3 min. The size of tissue block was < 1 mm^3^. The 1mm^3^ tissue blocks were transferred into an EP tube with fresh TEM fixative for further fixation, which was fixed at 4 °C for preservation and transportation. Microstructure of tissue was observed after standard TEM sample preparation procedure with a TEM of HITACHI HT7800/HT7700. For measuring the G‐ratio, the G‐ratio was calculated by inner diameter of the myelin sheath/outer diameter of the myelin sheath, with at least 100 axons being analyzed per animal.

### 2.6 Intracisternal CSF Tracer Infusion

Fluorescent CSF tracer (Dextran‐Texas Red, 3 kDa, Invitrogen D3328) was formulated in artificial CSF at a concentration of 1% weight by volume. Mice were anesthetized with 1.25% tribromoethanol at 50 g/mL and the CM were exposed. A 30‐gauge needle filled with the tracer was inserted into the CM. Five microliters of CSF tracer was infused at a rate of 2.5 μL/min for 2 min. The visualization of tracer movement from the cisternal compartments into the brain parenchyma were performed by transcranial imaging (Leica M205 FA). The animal skin was removed from the top of the head to expose the skull and the animal was placed under the microscope. Image acquisition was once 5 min on the Cy3 channel, from 10 to 30 min. After transcranial imaging in 30 min, the animals were killed and perfused with 20 mL of PBS and 20 mL of 4% paraformaldehyde. The brain was fixed overnight by immersion in 4% paraformaldehyde, followed cryoprotection in 30% sucrose in PBS for 2 days at 4°C. Coronal brain slices (100 μm) were cut on a frozen microtome (Leica CM1950). Tracer influx into the brain was quantified using ImageJ software (NIH). Quantification of Dextran coverage was performed on cerebral cortex of the whole brain by two independent researchers who were blinded of the genotype. Means of the data collected by the two researchers were used in statistical analyses.

### 2.7 Intracranial Evans Blue Injection

Anesthetized mice were placed on a stereotaxic frame (RWD Life science Co. Ltd. 68045). The skin was opened, and the mice were stereotaxically injected with 1 μL of 4% Evans blue into the right striatum a rate of 0.2 μL/min for 5 min (coordinates from Bregma: AP +0.6 mm; ML −2.0 mm; and DV +3.3 mm). After the end of the injection, needle was left in place for 5 min and then removed slowly to avoid backflush. Animals were kept under anesthesia from the start of the infusion to allow intraparenchymal movement of the tracers. After 90 min, blood was collected by 1 mL syringe rinsed with heparin. Then the animals were perfused with 20 mL of PBS and 20 mL of 4% paraformaldehyde. The blood was centrifuged at 10,000 g for 20 min, and the supernatant were measured at 620 nm to calculate the EB concentration. The brain was fixed overnight by immersion in 4% Paraformaldehyde, followed cryoprotection in 30% sucrose in PBS for 2 days at 4°C. Coronal brain slices (100 μm) were cut on a frozen microtome (Leica CM1950). The area of EB coverage were quantified using ImageJ software (NIH).

### 2.8 AAV Injection

Surgical procedures for intracranial AAV2/5 microinjections have been described previously [29]. In brief, mice were anesthetized and placed onto a stereotaxic frame. The skin was opened, and the mice were injected with 1 μL of AAVs into the right striatum at 0.2 μL/min (coordinates from Bregma: AP +0.6 mm; ML −2.0 mm; and DV +3.3 mm). Needle were withdrawn after 5 min and scalps were cleaned and sutured with sterile surgical sutures. Mice were allowed to recover in clean cages with ad libitum access to food and water. Subsequent experiments were performed 21 days after surgeries. AAV2/5 hGFAPS‐EGFP‐WPREs‐SV40pA and AAV2/5 hGFAPS‐EGFP‐P2A‐mAqp4‐WPRE‐SV40pA used in this study were purchased from PackGene Biotech. The mAqp4 coding sequence encoded the M1 isoform of AQP4.

### 2.9 Primary Mouse Astrocyte Culture

Primary astrocyte culture was prepared from the whole brains of NOTCH3‐WT or NOTCH3‐R170C mouse pups (P1) as described previously [30]. Briefly, brain tissue of mouse pups was isolated and transferred to ice‐cold astrocyte culture media (DMEM, high glucose +10% fetal bovine serum +1% Penicillin/Streptomycin). The meninges were removed the cortex were cut into small pieces. The tissue was then digested in 0.125% trypsin at 37°C for 15 min. The single cells were collected by centrifugation at 300 g for 5 min and seeded in 75 mm^2^ flasks with astrocyte culture media. Microglia and oligodendrocyte lineage cells were removed by shaking the culture at 200 rpm, 37°C for overnight. Astrocyte were enriched after removing the non‐attached cells.

### 2.10 Organotypic Slice Cultures

Organotypic brain slice were prepared from the cerebrum of postnatal Day 7 NOTCH3‐WT or NOTCH3‐R170C mouse pups as described previously [31]. Briefly, cerebrum of mouse pups was isolated and transferred to ice‐cold HBSS buffer. The meninges were cleared and the cerebrum were placed on the cutting stage of a McIlwain tissue chopper (CAVEY LAB, MTC‐21) and sliced into 500 μm thick sections. Then, the organotypic slice were transferred to cell culture insert (pore diameter = 0.4 μm) and cultured in 1 mL OSCs medium (36.7% Basal medium eagle [BME, Thermo Fisher Scientific], 36.7% Neurobasal‐A Medium [Thermo Fisher Scientific], 1% GlutaMAX [GIBCO], 0.033% insulin [Beyotime], and 0.5% P/S, 25% heat‐inactivated horse serum [GE Hyclone]). After 24 h, the OSCs medium was replaced by fresh medium followed by medium changes every 2 days up to Day 7.

### 2.11 Flow Cytometric Analysis

Flow cytometric analysis of primary astrocytes were performed with a flow cytometer (Agilent, NovoCyte Advanteon). Primary astrocytes were washed with PBS and subjected to surface labeling. The following surface markers were used: APC conjugated anti mouse CD31 (Biolegend 102,410, clone: 390, 1:400), APC conjugated anti mouse O4 (Miltenyi, 130‐119‐982, 1:200), Alexa Fluor 488 conjugated anti mouse GFAP (Biolegend 644,704, clone: 2E1.E9, 1:400), and PE‐Cyanine7 conjugated anti mouse TMEM119 (Invitrogen 25‐6119‐82, clone: V3RT1GOsz, 1:400). Data analysis was performed using FlowJo software (FlowJo, version 10.0, Ashland, OR).

### 2.12 Molecular Docking

The molecular interaction of NOTCH3, RUNX1, and CMYB were ensured using methods established in the literature [32]. The available crystal structures of the protein were downloaded from the Uniprot (https://www.uniprot.org). The three proteins were NOTCH3 (PDB ID: AF‐Q61982), RUNX1 (PDB ID: 1EAQ), and CMYB (PDB ID: 1H89). The files were saved in pdb format. Cluspro 2.0 docking server (https://cluspro.bu.edu/) [33, 34, 35] was used to dock the interaction between NOTCH3 and RUNX1 or RUNX1 and CMYB. After the docking prediction by Cluspro, the interaction was saved in pdb format and the PyMOL software (http://www.pymol.org/pymol) was used to create the 3D structures. Meanwhile, weighted score was obtained from Cluspro.

### 2.13 Immunofluorescence Staining

In in vivo experiments, mice were killed at indicated time points. After sufficient perfusion with 20 mL of PBS and 20 mL of 4% paraformaldehyde, brains were removed and cut into coronal sections (25 μm) on a frozen microtome. In in vitro experiments, astrocytes were seeded on coverslips coated with poly‐L‐lysine (Biosharp, BS005). After treatment, the cells were fixed with 4% paraformaldehyde. Brain sections or fixed astrocyte were washed and incubated with primary antibodies overnight in PBS containing 0.03% Triton‐X100 and 3% BSA. After wash, sections or cells were incubated with secondary antibodies for 1 h at room temperature. The following primary antibodies were used: mouse anti‐N3ECD (Sigma‐Aldrich, clone 1E4, 1:300), rabbit anti‐αSMA (Proteintech 14,395‐1‐AP, 1:100), rat anti‐CD31 (Bioscience 550,274, 1:50), rabbit anti‐AQP4 (Santa Cruz Biotechnology sc‐20,812, 1:500), mouse anti‐GFAP (CST 3670S, 1:500), rabbit anti‐RUNX1 (Affinity Biosciences AF6379, 1:300), rabbit anti‐CMYB (Affinity Biosciences AF6136, 1:300), mouse anti‐S100β (Sigma‐Aldrich S2532, 1:500), rabbit anti‐iba1 (Wako 019‐19,741,1:500), rabbit anti‐NEUN (Abcam ab177487, 1:500), mouse anti‐CC1 (Calbiochem OP80, 1:500), rabbit anti‐CDKN2A/p16INK4a (Abcam ab211542, 1:500), and rabbit anti‐p21(Affinity Biosciences AF6290, 1:300). The following secondary antibodies were applied: anti‐rat secondary antibody conjugated with Cy3 (Jackson ImmunoResearch Laboratories 112‐545‐003, 1:1000), anti‐rabbit secondary antibody conjugated with Cy3 (Jackson ImmunoResearch Laboratories 111‐165‐003, 1:1000), anti‐rabbit secondary antibody conjugated with 488 (Jackson ImmunoResearch Laboratories 111‐545‐003,1:1000), anti‐rabbit secondary antibody conjugated with Alexa Fluor 647 (Jackson ImmunoResearch Laboratories 111‐605‐003, 1:1000), anti‐mouse secondary antibody conjugated with Cy3 (Jackson ImmunoResearch Laboratories 115‐165‐003, 1:1000), anti‐mouse secondary antibody conjugated with 488 (Jackson ImmunoResearch Laboratories 115‐095‐003, 1:1000), anti‐mouse secondary antibody conjugated with Alexa Fluor 647 (Jackson ImmunoResearch Laboratories 115‐605‐003, 1:1000), and DAPI Fluoromount‐G (Abcam, ab104139) was applied to locate nucleus when indicated. Images were captured with a confocal microscopy (Leica TSC SP8) and processed with ImageJ software by a blinded observer for the unbiased counting of automatically recognized cells and mean fluorescent intensity calculation.

### 2.14 The Polarization of AQP4

Perivascular polarization of AQP4 was measured as described previously [36]. Briefly, the median immunofluorescence intensity and area of AQP4 was measured (total AQP4 area). Then the area which exhibited AQP4 immunofluorescence greater than or equal to perivascular AQP4 immunofluorescence (AQP4% area) was calculated. The none polarization was expressed as the percentage of the region that exhibited lower AQP4 immunoreactivity than the perivascular endfeet. The polarization of AQP4 was measured by AQP4% area/total AQP4 area.

### 2.15 Lentiviral Infection of Astrocyte

Overexpression of RUNX1 was fulfilled by inserting *Runx1* cDNA into the lentiviral transfer vector of TK‐PCDH‐copGFP‐T2A‐Puro. The constructed transfer vectors were transformed into DH5α *E. coli* and then isolated using the NucleoBond Xtra Midi EF (MN, 740420). A plasmid mixture containing psPAX2, pMD2.G, and the transfer vector was suspended in OPTI‐MEM and PEI 25K (Polysciences 23,966‐1) was applied as transfection reagent. The plasmids containing OPTI‐MEM was then added to 293T cells and allowed incubation for 6 h before switching to fresh culture medium. Supernatant was collected at 48 h after transfection and centrifuged at 3000 *g* for 10 min to further eliminate cell debris. The lentivirus containing medium was treated to astrocytes for 48 h to fulfill RUNX1 overexpression, whose efficiency was evaluated with western blot.

### 2.16 Western Blot

Protein was extracted with RIPA lysis buffer (Beyotime, P0013J) from the brain or astrocyte. A total amount of 40 μg protein of each sample was applied to western blot experiments. Western blot was performed with standard SDS‐polyacryamide gel electrophoresis method and HiPer ECL Western HRP Substrate (Mei5bio, MF074). The following primary antibodies were used: rabbit anti‐AQP4 (Proteintech 16,473‐1‐AP, 1:1000), rabbit anti‐RUNX1 (Affinity Biosciences AF6379, 1:1000), rabbit anti‐CMYB (Proteintech 17,800‐1‐AP, 1:1000), rabbit anti‐NOTCH3 (Proteintech 55,114‐1‐AP, 1:1000), mouse anti‐GAPDH (Proteintech 60,004‐1‐Ig, 1:10000), mouse anti‐β‐ACTIN (Proteintech 66,009‐1‐Ig, 1:10000), and mouse anti‐LAMIN B1 (Proteintech 66,095‐1‐Ig, 1:10000). Immunoreactivity was assessed with ImageJ software.

### 2.17 Dual‐Luciferase Reporter Assays

Overexpression of CMYB was fulfilled by inserting *Cmyb* cDNA into the export vector of pEGFP‐N1. DNA fragments of *Aqp4* promoter were synthesized by Sangon Biotech and then subcloned into pGL3‐Basic firefly luciferase reporter vector. Fifty nanograms of *Cmyb* vectors (*Cmyb* overexpressing vector or control), 50 ng firefly luciferase reporter vectors, and 5 ng pRL Renilla Luciferase Control Reporter Vector were co‐transfected into HEK293T cells in 96‐well plates. Fluc and Rluc activities were measured 48 h later with the Dual‐Luciferase Reporter Assay System (Promega, E1910) according to the manufacturer's instructions. The relative luciferase activity was calculated through dividing Fluc activity by individual Rluc activity and then normalizing to control.

### 2.18 Blue Native PAGE (BN‐PAGE)

Protein was extracted with RIPA lysis buffer (Beyotime, P0013J) from NOTCH3‐WT or NOTCH3‐R170C astrocytes. The lysis was performed on ice for 30 min and the samples were then centrifuged at 16,000 *g* for 10 min. The protein content of the supernatant was diluted by 2X Blue Native PAGE Sample Buffer (Sangon Biotech, C506055‐0005). Eight percent polyacrylamide native gradient gels were prepared (Sangon Biotech, C631101‐0100). The running buffers were the anode buffer and the blue cathode buffer (Sangon Biotech, C506045‐0001). Electrophoresis was performed at 50 mV at the first 30 min at 12°C, and then at 5 mA for 5 h. The lanes from the first dimension were cut into individual strips and the strips were equilibrated in denaturation buffer (1% SDS and 1% β‐mercaptoethanol) for 1 h at room temperature and placed into a 2D SDS–PAGE of the same thickness. The second‐dimension run was performed as described earlier.

### 2.19 Real Time Polymerase Chain Reaction (RT‐PCR)

Total RNA from cells or organotypic brain slice was extracted with RNA Quick Purification kit (ESscience, RN001) according to the manufacturer's instructions. A total of 1 μg RNA (OD260nm/280 nm = 1.8–2.2) was applied to the first strand cDNA synthesis in a 40 μL system using Fast Reverse Transcription kit (ESscience, RT001). Real time polymerase chain reaction (RT‐PCR) was performed on a QuantStudio 5 (ABI) quantitative PCR machine using SYBR Green qPCR Mix (GDSBio, P2092a) with 0.5 μL of the synthesized cDNA in each reaction with. The following program was performed: 95°C for 30s; 95°C for 5 s and 60°C for 34 s, repeated for 40 cycles; 95°C for 15 s, 60°C for 1 min, and 95°C for 15 s (Melt curve). Primers used in the study are listed in Table S2. Delta CT log2 (compared with the CT value of *Actb*) was calculated and normalized to the means of control group.

### 2.20 Immunoprecipitation (IP)

Protein of 5 × 10^6^ astrocytes was extracted with 1 mL of RIPA lysis buffer (Beyotime, P0013J). Antibodies (2 μg per sample) were applied to the IP system and incubated overnight at 4°C on a shaker. Pierce protein A/G magnetic beads (Thermo Fisher, Carlsbad, CA, USA, 88802) were then added into the system (5 μL per sample). The IP system was then incubated in a rotator for additional 2–4 h at 4°C. After three washes with 1 mL RIPA lysis buffer, 100 μL of HCL‐Glycine (pH = 2.0) was applied to elute the precipitated protein. The sample was then subjected to western blot experiments. The following antibodies were used: rabbit anti‐CMYB (Proteintech 17,800‐1‐AP), rabbit anti‐NOTCH3 (Proteintech 55,114‐1‐AP), and normal rabbit IgG (CST 2729).

### 2.21 Senescence‐Associated Beta Galactosidase (SA‐β‐Gal) Staining

SA‐β‐Gal staining was done using SA‐β‐Gal staining kit (Beyotime C0602) according to the manufacturer's instructions. The detection of SA‐β‐Gal activity is typically facilitated by the substrate called 5‐bromo‐4‐chloro‐3‐indolyl β‐D‐galactopyranoside (X‐Gal). X‐Gal, a derivative of β‐D‐galactoside, generates an intense blue reaction product in the presence of β‐galactosidase. During cellular senescence, the activity of SA‐β‐Gal is elevated. Consequently, under optimal conditions, enhanced enzyme activity can be visualized through the utilization of X‐Gal as a substrate. Brain slices were fixed and then stained with the staining solution overnight at 37°C in a non‐CO_2_ incubator. Senescent cells were identified as blue‐stained cells under light microscopy (Nikon Ti2‐U).

### 2.22 Single Cell RNA Sequencing

Mice (16w of aged, *n* = 2 in each group) were anesthetized with 1.25% tribromoethanol and perfused with sufficient ice PBS. Cerebellum and olfactory bulb were removed and brain were gently dissected in PBS. Then, whole brains were digested, myelin was removed, and single cell suspension were prepared for single cell sequencing. Next, the cells were droplet separated using Chromium Single Cell 3′ v3 Reagent Kit with the 10x Genomics microfluidic system creating cDNA library with individual barcodes for individual cells. Barcoded cDNA transcripts from stroke mice were pooled and sequenced using the novaseq 6000 Sequencing System. Demultiplexing, alignment to the GRCh38 reference with the *Mus musculus* genome (GRCm38) by the STAR aligner v2.5.1b with default settings, and quantification of sequencing reads for each sample were performed using Cell Ranger (Version 4.0.0) with the default parameter. The filtered gene‐barcode matrices for single cells were analyzed using Seurat (V4) following the tutorial at https://satijalab.org/seurat/. Cells were filtered out with more than 5% of mitochondrial genes and fewer than 200 or greater than 5000 detected genes, and more than 30,000 unique molecular identifiers (UMIs) per sample. The quality of the filtered cells was normalized using the Seurat (V4) package. Gene counts for each cell were divided by the total counts for each cell and multiplied by the scale.factor (default is 10,000), then natural‐log transformed using log1p(), which the equation was $y=\frac{\log1p\left(i\right)}{\mathrm{sum}\left(i\right)}\times \text{scale}.\text{factor}$, where i denoted gene count for each cell, using the NormalizeData() function in Seurat. After filtering and normalization, samples were integrated directly, and performed dimensionality reduction and clustering using the Seurat V4 in *R*. Variable genes (genes = 4000) were selected through FindVariableFeatures() function and those variable genes were assigned onto a low‐dimensional subspace using principal component analysis through RunPCA() function. Seurat functions FindNeighbors() and FindClusters() were used to assign subtypes with the top 50 principal components. To visualize the results, Uniform Manifold Approximation and Projection (UMAP) was performed with Harmony embeddings. Differentially expressed genes were calculated with the FindMarker() function in Seurat (Wilcoxon rank‐sum test). For celltype, we manually annotated clustered cells into eight subgroups based on differentially expressed genes, including astrocytes (*Aqp4; Gja1; Ndrg2; Gfap*), neurons (*Snap25; Stmn2; Rbfox3*), microglia (*Cx3cr1*; *Tmem119*), oligodendrocytes (*Plp1*; *Mag*; *Mbp*), oligodendrocyte precursor cells (OPC) (*Pdgfra*; *Pcdh15*; *Olig2*), vascular cells (endothelial cells (*Cldn5*; *Vtn*; *Pecam1*), pericytes (*Pdgfrb*; *Acta1*), *SMC* (*Abcc9*; *Acta2*; *Pdgfrb*; *Rgs5*; *Tagln*)), perivascular macrophages (PBM) (*Ma4a7*; *Mrc1*), and other cells. Other cells referred to individuals that may contain two cells. We queried the top differentially expressed genes of each cluster in a literature search to annotate the clusters. Differentially expressed genes were visualized in UMAP or violin plots.

### 2.23 Statistical Analysis

A Kolmogorov–Smirnov test of normality was performed before the parametric analysis of Student's *t* test, one‐way ANOVA, and two‐way ANOVA. GraphPad Prism software (version 9.0) and SPSS Statistics (version 27.0.1) was used for statistical analysis. A Kolmogorov–Smirnov test of normality was performed. Student's *t* test: Unpaired parametric *t* test (two‐tailed) was performed in data comparison of two groups. Error bar represents standard error of mean (SEM). One‐way ANOVA: No matching ANOVA was performed in data comparison of three groups or more. Result was corrected for multiple comparisons using statistical hypothesis testing (Dunnett). Error bar represents SEM. Two‐way ANOVA: Two‐way ANOVA was performed in data on a quantitative dependent variableat multiple levels of two categorical independent variables. Spearman correlation: Correlation between every pair data sets was computed with nonparametric Spearman correlation coefficients. ROC analysis: In the current study, ROC analysis has been used to evaluate the diagnostic efficacy of EPVS to distinguish brain atrophy, or to evaluate whether including EPVS, in addition to the GCA score could increase the distinguishing efficacy for CADASIL. Probabilities were calculated in SPSS and subjected to GraphPad Prism for ROC curve description.

### 2.24 Study Approval

The clinical and the animal experimental studies were approved by the Medical Ethics Committee of the Third Affiliated Hospital of Sun Yat‐sen University and the Animal Care and Use Committee of Sun Yat‐sen University respectively. All participants had been given the informed consent according to the principles illustrated in Declaration of Helsinki.

## 3 Results

### 3.1 Brain Atrophy in CADASIL Patients Is Associated With PVS Enlargement

**FIGURE 1:**
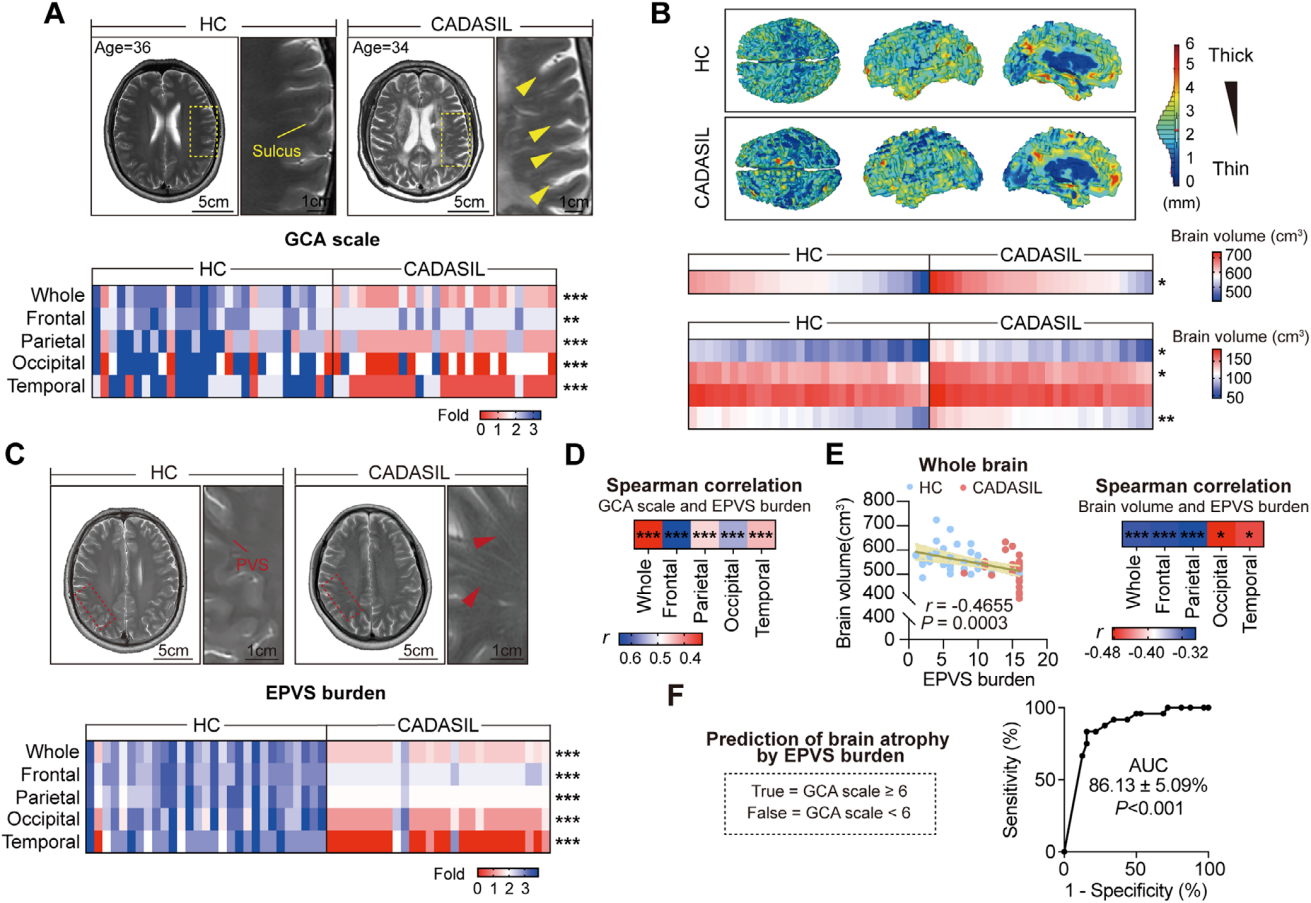
MRI‐based brain atrophy and perivascular space (PVS) enlargement in CADASIL patients and healthy controls. (A, B) Brain atrophy of CADASIL patients (*N* = 27) and HC (*N* = 29) was evaluated with GCA scale (A) and brain volume (B) based on MRI data. (A) The GCA scale was assessed according to sulci dilatation. Upper: Representative MR images of HC and CADASIL patient. Yellow arrowheads indicated the normal width of sulci in HC and the widen sulci in patient. Lower: Heatmap showing the GCA scale of each recruited individual (normalized to HC). **p < 0.01 and ***p < 0.001; by nonparametric test. (B) Brain volume (total gray matter volume) was quantified with SPM12 package and CAT12 toolbox in MATLAB based on MR images. Upper: Representative 3D‐reconstructed images by CAT12 toolbox showing reduced brain volume of CADASIL patients. Lower: Heatmap showing brain volume of each recruited individual. *p < 0.05 and **p < 0.01; by Student's *t* test. (C) Representative MR images showing PVS of HC and CADASIL patient. Red arrowheads indicated PVS, which is linear fluid‐filled space following the course of penetrating vessel of cerebrospinal fluid signal. Red dashed lines outlined the enlarged PVS in the CADASIL patient. Lower: Heatmap showing the EPVS burden in each recruited individual. ***p < 0.001; by nonparametric test. (D) Spearman correlation analysis of EPVS burden and GCA scale in the cohort. (E) Spearman correlation analysis of EPVS burden and brain volume in the cohort. *p < 0.05 and ***p < 0.001. (F) Prediction efficacy of EPVS burden for moderate‐to‐severe brain atrophy (GCA scale ≥ 6) in the recruited cohort with ROC analysis.

To investigate the brain aging status and glymphatic function in CADASIL, a total of 27 patients and 13 age‐ and sex‐matched healthy controls (HC) were recruited in the study. Inclusion process is shown in Figure S1 and cohort demographics is displayed in Table S1. Consistent to previous reports [37, 38], MRI data of our cohort revealed that CADASIL patients exhibited increased global cerebral atrophy (GCA) scale (Figure 1A) and reduced brain volume (Figure 1B), suggesting the advanced brain senescence in CADASIL patients. In accordance with previous reports [19, 20], CADASIL patients in the current cohort showed remarkable EPVS in various brain lopes (Figure 1C), which indicated glymphatic impairment. To be noticed, EPVS burden was positively associated with GCA scale as revealed by Spearman correlation analysis (Figure 1D) and was negatively associated with brain volume in multiple linear regression analysis (Figure 1E). Moreover, EPVS burden showed pronounced predicting efficacy for moderate‐to‐severe brain atrophy (GCA scale ≥ 6) (Figure 1F) and could improve the predicting efficacy of GCA scale (Figure S2). These results illustrate that glymphatic dysfunction contributes to the advanced brain senescence in patients with CADASIL.

### 3.2 Glymphatic Influx and Efflux Are Both Retarded in CADASIL

**FIGURE 2:**
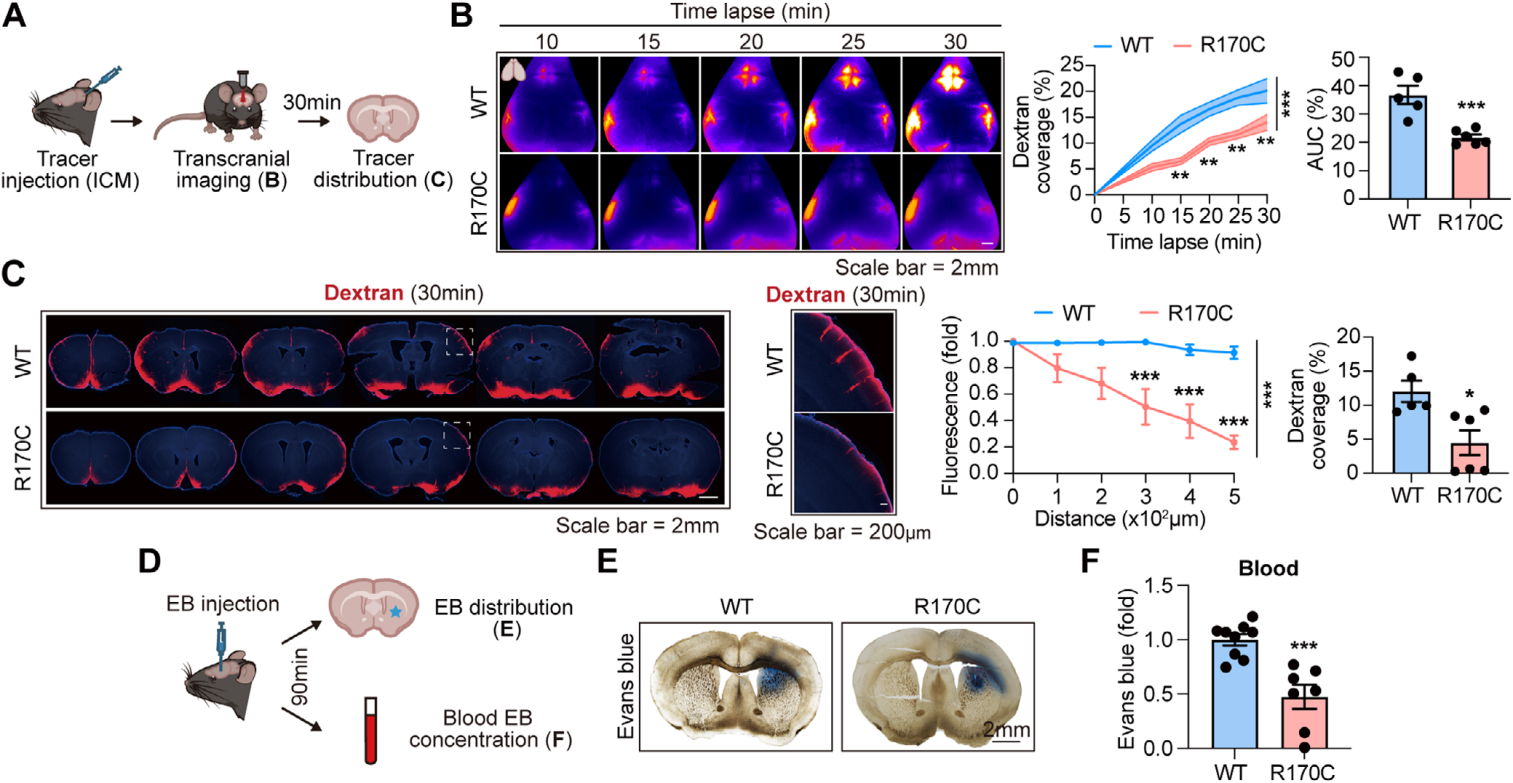
Evaluation of glymphatic function with NOTCH3‐R170C models. (A–C) The fluorescent tracer of 3 kDa Dextran‐Texas Red was injected into the CSF pool through cisterna magna (CM) (10 mg/mL in 5 μL) of NOTCH3‐R170C or ‐WT mice (16w of age) to evaluate glymphatic influx activity. (A) Experimental design for glymphatic influx assessment. (B) Dynamic Dextran coverage in brain as assessed with live imaging (through skull) at 0–30 min after injection. *N* = 5 in NOTCH3‐WT group and *N* = 6 in NOTCH3‐R170C group. **p < 0.01 and ***p < 0.001; by two‐way ANOVA (mean ± SEM). Area under curve (AUC) was calculated. ***p < 0.001, by Student's *t* test (mean ± SEM). (C) At 30 min after Dextran injection, mice were killed and the coronal brain sections (100 μm) were subjected to microscopy. Fluorescence intensity of Dextran at indicated distance from brain surface was calculated (Normalized to each brain surface). *N* = 5 in NOTCH3‐WT group and *N* = 6 in NOTCH3‐R170C group. ***p < 0.001; by two‐way ANOVA (mean ± SEM). Dextran coverage in the whole brain was analyzed. *p < 0.05, by Student's *t* test (mean ± SEM). (D–F) A total of 1 μL EB (960 Da, 40 mg/mL) was injected into striatum and blood EB level at 90 min after injection was recorded to evaluate glymphatic efflux function. (D) Experimental design for glymphatic efflux assessment. (E) Representative images for EB covering area. (F) EB level in plasma at 90 min after injection (620 nm, through plate reader) as calculated by fold change to NOTCH3‐WT mice. *N* = 9 in NOTCH3‐WT group and *N* = 7 in NOTCH3‐R170C group. ***p < 0.001; by Student's *t* test (mean ± SEM).

To further evaluate the impact of NOTCH3 mutation on glymphatic functions in CADASIL, homozygous *Notch3* ^R170C+/+^ mutant mice (NOTCH3‐R170C) with C57/BL6 background were used as animal models and glymphatic activities were analyzed. NOTCH3‐R170C mice are a knockdown model that contains a cysteine mutation with the insertion of codon 170 in the mouse *Notch3* gene, corresponding to the human Arg169Cys mutation. In consistence with previous reports [28, 39, 40, 41, 42], deposition of granular osmiophilic material (GOM) on brain blood vessel of NOTCH3‐R170C mice was evident (Figure S3A), Besides, accumulation of NOTCH3 extracellular domain (ECD) in brain vasculature of the NOTCH3‐R170C mice was recorded, which further validated the effectiveness of the model (Figure S3B). Glymphatic influx that mediates the infusion of CSF into brain parenchyma was first evaluated (Figure 2A–C). The fluorescent tracer of 3 kDa Dextran‐Texas Red (10 mg/mL in 5 μL) was injected into the CSF pool through cisterna magna (CM). Movement of the tracer was dynamically recorded by trans‐skull lived imaging for 30 min (Figure 2A). We found that the Dextran penetration from CSF to brain ISF was slow in NOTCH3‐R170C mice (Figure 2B). At 30 min after Dextran injection, the mice were killed and the brains were subjected to frozen section (100 μm) and microscopy. We recorded diminished Dextran infusion in NOTCH3‐R170C mice (Figure 2C). To be noticed, heterozygous *Notch3* ^R170C+/−^ mice displayed similar impairment of glymphatic influx compared with their homozygous littermates (Figure S3C).

Glymphatic efflux, which drains out ISF to the periphery, was then analyzed (Figure 2D–F) [43, 44]. Evans blue (EB), a small molecule (960 Da) that diffuses through glymphatic system and binds to albumin in blood, was injected into striatum. At 90 min after injection, EB distribution in brain and excretion to blood were analyzed (Figure 2D). We found that although EB covering area was comparable between NOTCH3‐R170C and ‐WT groups (Figure 2E), serum EB level was prominently decreased in the CADASIL models (Figure 2F). Similarly, impairment of glymphatic efflux was recorded in heterozygous *Notch3* ^R170C+/−^ mice, though at a lower degree compared to their homozygous littermates (Figure S3D,E). Notably, at 180 min after intra‐cisterna magna injection, the amount of Dextran that entered the brain (Figure S3F) or drained to the blood (Figure S3G) was similar between CADASIL models and WT controls, indicating that although trans‐glymphatic influx and efflux were delayed, the processes were not completely blocked.

### 3.3 NOTCH3 Mutation Leads to Diminished AQP4 Expression in Astrocyte and Glymphatic Failure

**FIGURE 3:**
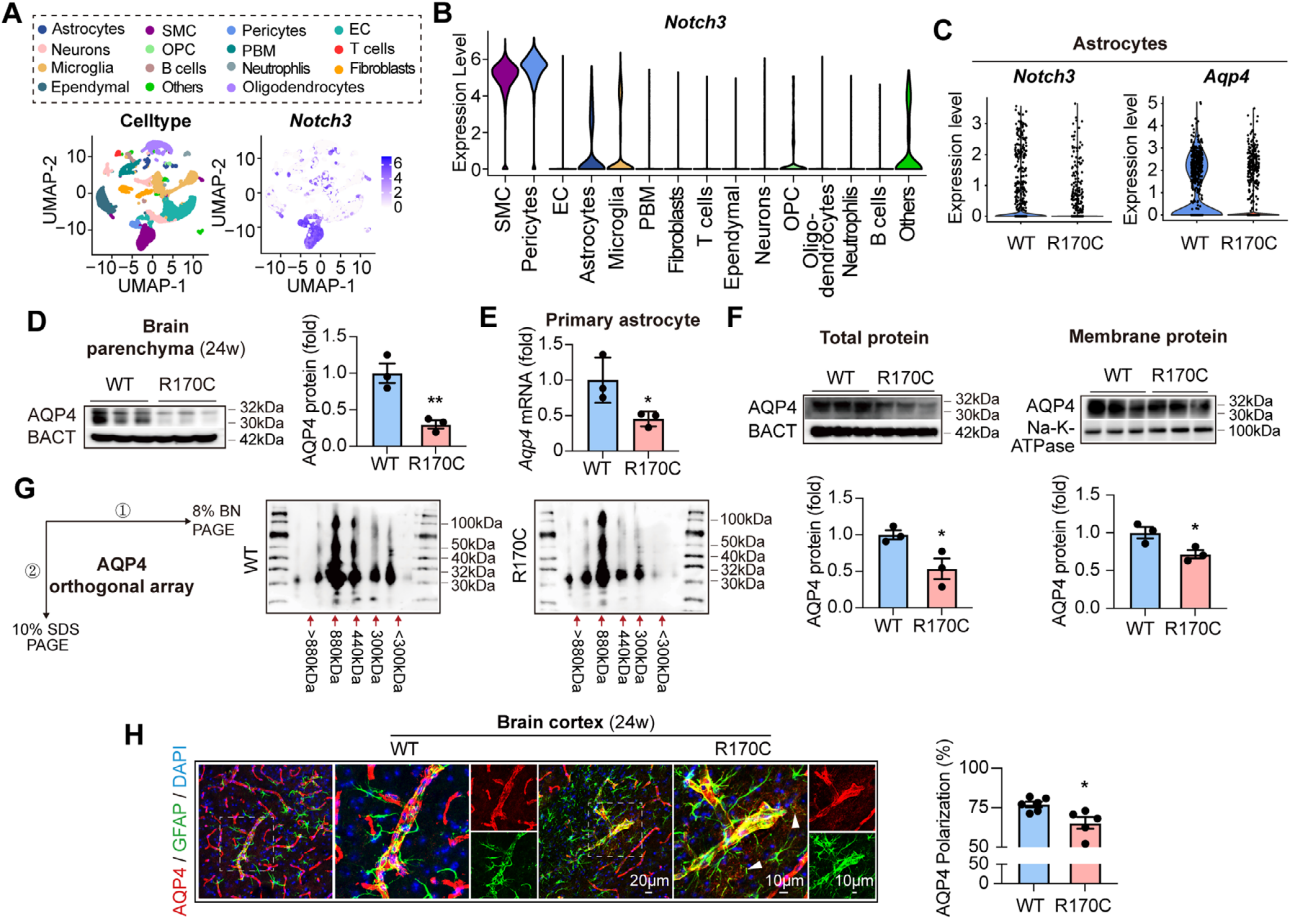
Diminished AQP4 expression in NOTCH3‐R170C astrocyte. (A–C) Brain cells of NOTCH3‐R170C and ‐WT mice (16 weeks of age) were subjected to single cell RNA sequencing (scRNAseq). (A) Left: Merged Uniform manifold approximation and projection (UMAP) plot representing 15 color‐coded cell clusters identified in the combined single‐cell transcriptomes. Cluster names were manually assigned. Right: Feature plots of *Notch3*. (B) Expression of *Notch3* among brain cells. (C) Violin plots of *Notch3* and *Aqp4* expression in astrocytes. (D) Brain protein of NOTCH3‐R170C or ‐WT mice (24 weeks of age) was subjected to western blot to evaluate AQP4 expression. *N* = 3 in each group. **p < 0.01; by Student's *t* test (mean ± SEM). (E, F) Primary NOTCH3‐R170C and ‐WT astrocyte cultures were prepared. (E) *Aqp4* mRNA level was evaluated with RT‐PCR. (F) AQP4 protein expression in whole cell and plasma membrane of astrocytes was assessed with western blot. Experiments were repeated for three times. *p < 0.05; by Student's *t* test. Difference of means (± SEM) is displayed. (G) Primary NOTCH3‐R170C and ‐WT astrocyte cultures were subjected to BN page and then 2D SDS page to evaluate the orthogonal arrays of particles (OAPs) of AQP4. (H) Coronal brain sections of NOTCH3‐R170C or ‐WT mice (24 weeks of age) were subjected to immunostaining of GFAP (green) and AQP4 (red). The AQP4 polarization was calculated by AQP4 perivascular endfeet area. White arrowheads indicated the depolarized AQP4 distribution. *N* = 6 in NOTCH3‐WT group and *N* = 5 in NOTCH3‐R170C group. *p < 0.05; by Student's *t* test (mean ± SEM).

AQP4 on astrocyte endfeet mediates CSF‐ISF exchange and conducts ISF movement, thus is regarded as the key coordinator of glymphatic system [44]. Moreover, previous researches have reported that NOTCH signaling pathway is activated in astrocytes [45, 46, 47]. Since both glymphatic influx and efflux were impaired in the CADASIL models, we speculated that AQP4 expression and/or distribution were dysregulated due to NOTCH3 mutation. To explore the hypothesis, NOTCH3‐R170C and ‐WT mice (16 weeks of age) were subjected to single cell RNA sequencing (scRNAseq). We documented that *Notch3* was expressed in astrocytes beside pericytes and smooth muscle cells (Figure 3A,B), which was decreased in NOTCH3‐R170C mice (Figure 3C). Furthermore, we found *Notch3* expression in astrocytes across various scRNAseq datasets from neurologic diseases, including aging, LPS‐induced inflammation, stroke, Alzheimer disease, and experimental autoimmune encephalomyelitis (EAE) [48, 49, 50, 51]. To be noticed, A*qp4* transcription was downregulated in NOTCH3‐R170C astrocytes (Figure 3C). The AQP4 gene encodes two mRNA isoforms with different translation initiating methionines, M1 or M23 [52]. Thus, AQP4 protein is expressed as two major isoforms of 32 kDa (AQP4‐M1) and 30 kDa (AQP4‐M23). Performing western blot, we found that both AQP4‐M1 and ‐M23 expression were diminished in NOTCH3‐R170C brain in vivo at both 16 weeks (Figure S4A) and 24 weeks (Figure 3D). AQP4 expression could be affected by multiple factors including the interactions between astrocytes and blood vascular cells [53]. To study whether the diminished AQP4 expression in CADASIL was a direct result of NOTCH3 mutation in astrocyte or a secondary consequence of NOTCH3 mutation in other brain cells, astrocytes were cultured from NOTCH3‐R170C or ‐WT mice (Figure S5A). In consistence with the in vivo data, primary NOTCH3‐R170C astrocyte cultures displayed downregulated AQP4 transcription (Figure 3E) and translation of AQP4‐M1 and ‐M23 (Figure 3F). AQP4‐M1 and ‐M23 are organized in the plasma membrane as heterotetramers, forming orthogonal arrays of particles (OAPs) on the astrocyte endfeet. The size of AQP4 OAPs ranges from several MDa to ~400 kDa in the brain, depending on the ratio of AQP4‐M1 to AQP4‐M23. AQP4‐M23 alone forms large size OAPs, whereas AQP4‐M1 alone is unable to form OAPs. When coexpressed, they form OAPs of intermediate size [54, 55]. Normal arrangement of AQP4 OAPs on astrocyte endfeet are indispensable for glymphatic transportation [56]. In the current study, BN/SDS–page was performed to examine the membrane organization of AQP4‐OAPs (Figure 3G). Plasma membrane proteins from primary NOTCH3‐R170C or ‐WT astrocytes were isolated and first separated in native conditions on an 8% acrylamide gradient blue native gel (BN‐page) then subjected to a 2D 10% acrylamide gel with sodium dodecyl sulfate (SDS‐page). AQP4 immunoblot performed on transferred proteins after 2D BN/SDS‐page revealed the presence of several distinct spots corresponding to AQP4 at 30–32 kDa at > 880 kDa, 880 kDa, 440 kDa, 300 kDa, and < 300 kDa containing M1 and M23 isoforms with variable expression levels (Figure 3G). Densitometric analysis was performed to determine the relative abundance of the 30 and 32 kDa isoforms in the seven AQP4 pools in NOTCH3‐WT and ‐R170C astrocytes, which turned out to be similar (statistics not shown). The results indicate that the structure of AQP4 OAPs is not affected by NOTCH3 mutation in CADASIL.

As for AQP4 distribution along brain blood vessel, no abnormality was recorded at 16 weeks (Figure S4B). Meanwhile, transcription of DAC (dystrophin‐associated complex) genes (*Dag1*, *Dtna*, *Dmd*, *and Snta1*) (Figure S5B) and gap junction protein genes (*Gja1* and *Gja6*) (Figure S5C), which are implicated in the regulation of AQP4 distribution [44], was comparable between NOTCH3‐R170C and ‐WT astrocytes in vitro. However, perivascular polarization of AQP4 was disturbed at 24 weeks (Figure 3H), which was probable a secondary alteration. These data illustrate that NOTCH3 mutation results in reduced AQP4 expression and depolarized AQP4 distribution, but does not affect AQP4 spatial configuration.

### 3.4 NOTCH3 Mutation Downregulates RUNX1‐CMYB‐AQP4 Signaling in Astrocyte

**FIGURE 4:**
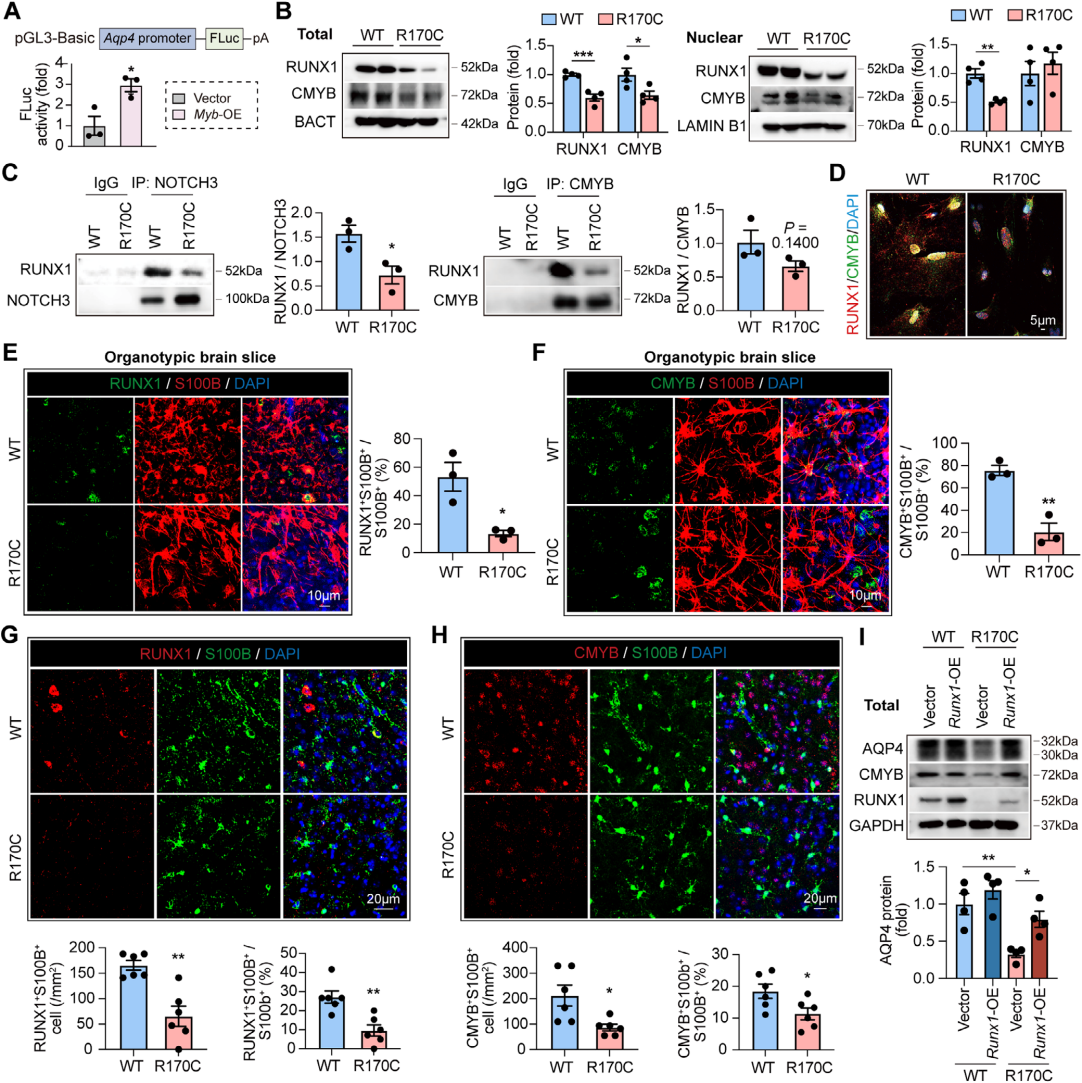
Activation of RUNX1‐CMYB‐AQP4 signaling is downregulated in NOTCH3‐R170C astrocyte. (A) Dual‐luciferase reporter assays showing the effect of CMYB activation on *Aqp4* transcription in HEK293T cells. Experiments were repeated for three times. *p < 0.05; by Student's *t* test (mean ± SEM). (B) Total or nucleus protein of NOTCH3‐R170C and ‐WT astrocytes were subjected to western blot. Protein level was normalized to BACT or LAMIN B1 expression. Experiments were repeated for four times. *p < 0.05, **p < 0.01, and ***p < 0.001; by Student's *t* test (mean ± SEM). (C) Protein was extracted from NOTCH3‐R170C or ‐WT astrocyte then subjected to immunoprecipitation (IP) with anti‐NOTCH3 or anti‐CMYB antibodies. IP products were analyzed with western blot to examine the interaction of NOTCH3 and RUNX1 or RUNX1 and CMYB, respectively. Experiments were repeated for 3 times. *p < 0.05; by Student's *t* test (mean ± SEM). (D) Immunostaining of CMYB (green) and RUNX1 (red) with NOTCH3‐R170C or ‐WT astrocyte. (E, F) NOTCH3‐R170C or ‐WT OSCs were labeled with RUNX1 (green) and S100B (red) (E) or CMYB (green) and S100B (red) (F), respectively. Percentage of RUNX1^+^ or CMYB^+^ cells among S100B^+^ astrocytes was calculated. Experiments were repeated for three times. *p < 0.05, **p < 0.01; by Student's *t* test (mean ± SEM). (G–H) Coronal brain sections of NOTCH3‐R170C or ‐WT mice (16 weeks of age) were subjected to immunostaining of RUNX1 (red) and S100B (green) (G) or CMYB (red) and S100B (green) (H). *N* = 6 in each group. *p < 0.05, **p < 0.01; by Student's *t* test (mean ± SEM). (I) RUNX1 was overexpressed in NOTCH3‐R170C or ‐WT astrocytes with lentiviral vectors carrying *Runx1* cDNA (*Runx1*‐OE) which were then subjected to western blot. Protein level was normalized to GAPDH expression. Experiments were repeated for four times. *p < 0.05, **p < 0.01; by one‐way ANOVA (mean ± SEM).

We next explored the mechanistic link between NOTCH3 mutation and diminished AQP4 expression. As revealed by signaling pathways projection (SPP, signalingpathways.org), the transcriptional factor (TF) of CMYB binds to *AQP4* gene. With dual‐luciferase reporter system, we confirmed that CMYB promoted AQP4 transcription (Figure 4A). According to previous report, CMYB activation is under the control of NOTCH‐RUNX1 signaling [11]. Performing molecular docking, we found certain interactions of NOTCH3‐RUNX1 and RUNX1‐CMYB‐DNA (Figure S6A). These data suggest that decreased AQP4 expression might be resulted from malfunction of NOTCH3‐RUNX1‐CMYB pathway in CADASIL. As revealed by scRNAseq, transcription of *Runx1* was unaffected in NOTCH3‐R170C astrocytes while expression of the CMYB coding gene *Myb* was decreased (Figure S6B). Western blot showed downregulated RUNX1 and CMYB protein level and reduced translocation into the nucleus in NOTCH3‐R170C astrocytes (Figure 4B). Accordingly, NOTCH3 mutation led to reduced NOTCH3‐RUNX1 and RUNX1‐CMYB interaction (Figure 4C). Performing immunostaining, we observed that intra‐nucleus distribution of RUNX1 and CMYB was downregulated in NOTCH3‐R170C astrocytes in vitro (Figure 4D), ex vivo (Figure 4E,F), and in vivo (Figure 4G,H), indicating the downregulation of signaling. To be noticed, overexpressing RUNX1 in NOTCH3‐R170C astrocytes restored the activity of RUNX1‐CMYB signaling (Figure 4I; Figure S6C,D) as well as AQP4 level (Figure 4I). These results reveal that AQP4 is the downstream target of NOTCH3‐RUNX1‐CMYB pathway. NOTCH3 mutation suppresses RUNX1‐CMYB activation, which subsequently results in diminished AQP4 expression and impaired glymphatic functions.

### 3.5 Reinforcing AQP4 Expression in Astrocytes by AAV‐Based Gene Therapy Resumes Glymphatic Functions and Protects Against Brain Senescence in CADASIL

To evaluate the impacts of blocking AQP4‐dependent glymphatic drainage, AQP4 inhibitor of TGN‐020 was injected to WT mice (AQP4i, 5 mg/mL in 5 μL, i.cm, twice a week). In the AQP4i‐treated mice, glymphatic influx and efflux were impaired (Figure S7A) and neurons displayed increased γH2AX expression (Figure S7B), suggesting that glymphatic blockage directly causes neuronal injury. Nevertheless, AQP4i did not cause P16 or P21 increment in neurons, which are considered senescence markers (Figure S7C,D). Inhibition of glymphatic function could lead to accumulation of metabolic waste in the brain [57]. We have documented brain atrophy in CADASIL patients (Figure 1A,B), which demonstrates the senescence of CADASIL brain cells. We thus infer that although AQP4 hypo‐expression would not directly lead to brain cell senescence, the consequent glymphatic dysfunction could advance brain aging in long term.

**FIGURE 5:**
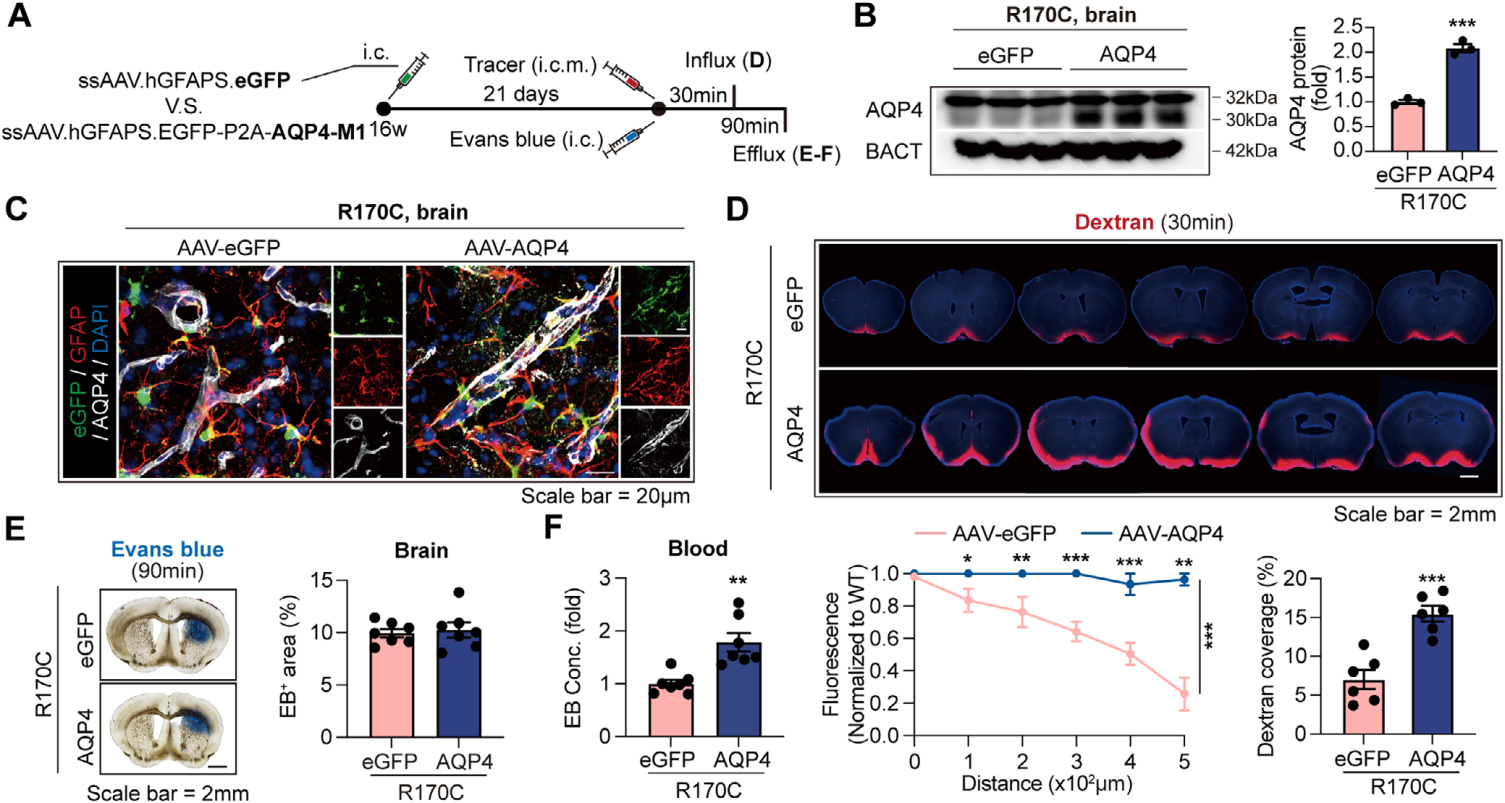
Astrocyte‐targeting AAV‐based AQP4 reinforcement resumes glymphatic functions in NOTCH3‐R170C mice. AAV‐AQP4 (ssAAV.hGFAPS.EGFP‐P2A‐AQP4‐M1) or AAV‐eGFP (ssAAV.hGFAPS.eGFP) was treated to NOTCH3‐R170C mice at the age of 16 weeks (Stereotactic injection into striatum). Mice were killed at 21 days after injection. (A) Experimental design. (B) Striatum of mice was collected and subjected to western blot. AQP4‐M1 (32kDa) and ‐M23 (30 kDa) expressions were analyzed (Normalized to BACT level). *N* = 3 in each group. ***p < 0.001; by Student's *t* test (mean ± SEM). (C) Coronal brain sections of mice were subjected to GFAP (red) and AQP4 (gray) staining. Green fluorescence of eGFP was recorded simultaneously. Representative images are displayed. (D) The fluorescent tracer of 3 kDa Dextran‐Texas Red was injected into the CSF (i.cm., 10 mg/mL in 5 μL) to evaluate glymphatic influx activity. At 30 min after injection, mice were sacrificed and the coronal brain sections were subjected to microscopy. Fluorescence intensity of Dextran at indicated distance from brain surface (Normalized to each brain surface) was calculated. *N* = 6 in each group. *p < 0.05, **p < 0.01 and ***p < 0.001; by two‐way ANOVA (mean ± SEM). Dextran coverage in the whole brain was analyzed. ***p < 0.001, by Student's *t* test (mean ± SEM). (E, F) A total of 1 μL EB (960 Da, 40 mg/mL) was injected into striatum and blood EB level at 90 min after injection was recorded to evaluate glymphatic efflux function. (E) EB covering area in brain. *N* = 7 in each group. (F) EB level in blood. *N* = 7 in each group. **p < 0.01; by Student's *t* test (mean ± SEM).

Considering that AQP4 expression is downregulated in the NOTCH3‐mutant CADASIL models, resulting in glymphatic dysregulation, we hypothesized that low AQP4 expression contributes to the advanced brain cell senescence in CADASIL and to re‐enhance AQP4 expression could reinforce glymphatic functions and ameliorate the senescence progression. To test the hypothesis, adeno‐associated virus (AAV) carrying *Aqp4* gene (AAV‐AQP4) that specifically targeted astrocytes was injected into the striatum of NOTCH3‐R170C mice at the age of 16 weeks (Figure 5A; Figure S8). Enhanced AQP4 expression in astrocytes of AAV‐AQP4‐treated CADASIL models was confirmed with western blot (Figure 5B) and immunostaining (Figure 5C, Figure S8B) at 21 days after injection. According to the western blot data, both the expression of AQP4‐M1 and ‐M23 was reenforced after the AAV treatment (Figure 5B). To evaluate the glymphatic influx efficiency after AAV‐AQP4 administration, the CSF tracer of Dextran (3 kDa Dextran‐Texas Red, 10 mg/mL in 5 μL) was injected through CM. Fluorescent intensity of Dextran at different distance from the brain margin at 30 min was calculated (Figure 2A). We found that Dextran permeation into brain parenchyma from CSF was significantly improved in the AAV‐AQP4‐treated NOTCH3‐R170C mice (Figure 5D), indicating the ameliorated glymphatic influx. Glymphatic efflux was assessed by measuring EB drainage efficiency from brain parenchyma to peripheral blood (Figure 2D). We found that AQP4 reinforcement in astrocytes promoted EB outflow from brain to blood in NOTCH3‐R170C mice (Figure 5F) in the premise of stable EB infusion (Figure 5E; Figure S9). In contrast, WT mice that received AAV‐AQP4 treatment did not show further improvement of glylmphatic functions (Figure S11A–C).

**FIGURE 6:**
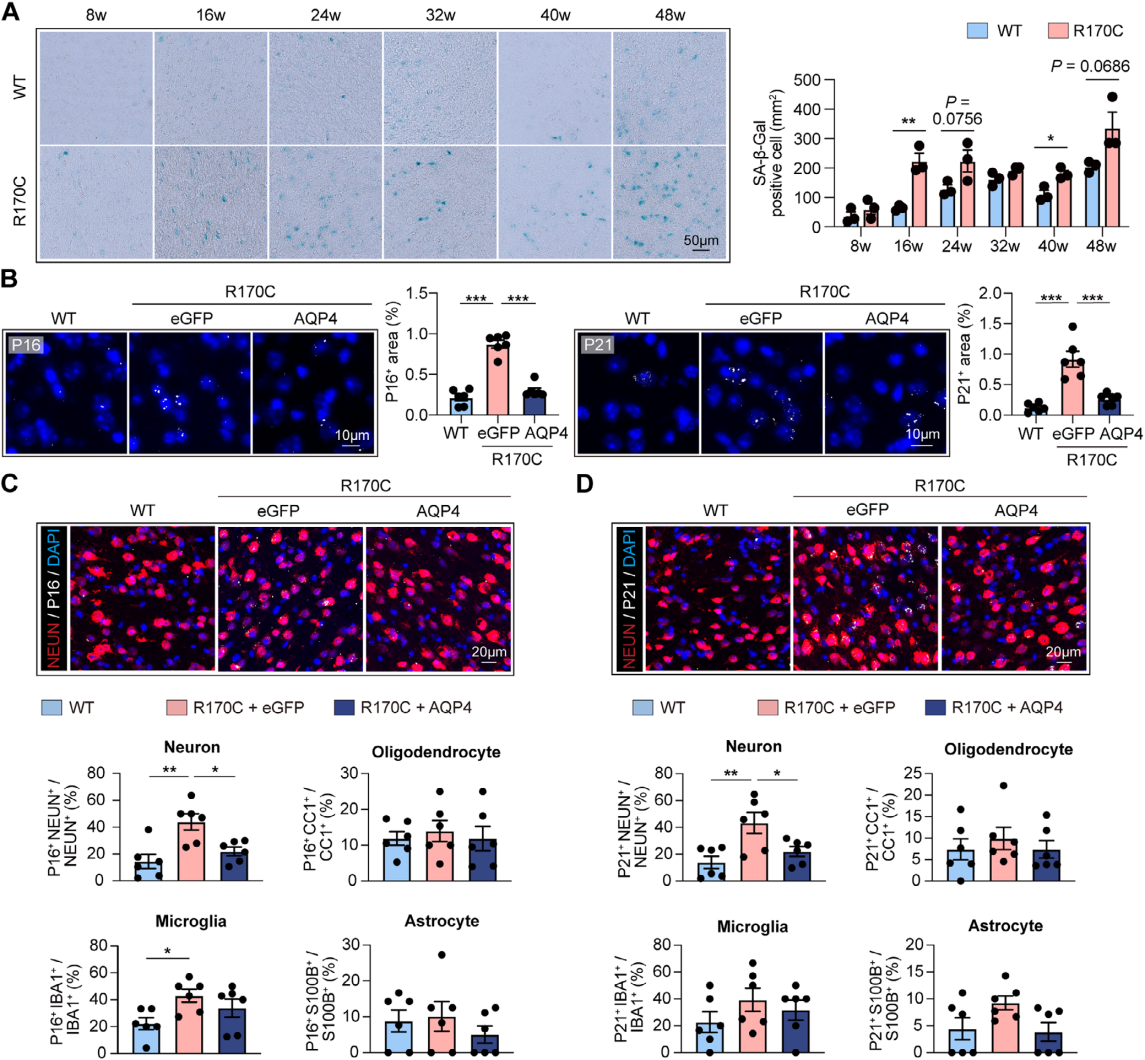
Astrocyte‐targeting AQP4 reinforcement by AAV‐based gene therapy protects against advanced brain senescence in NOTCH3‐R170C mice. (A) NOTCH3‐R170C and ‐WT mice were killed at indicated age and coronal brain sections were subjected to senescence‐associated β galactosidase (SA‐β‐Gal) staining. The number of SA‐β‐Gal‐positive cells were calculated. *N* = 3 in each group. *p < 0.05, **p < 0.01; by Student's *t* test (mean ± SEM). (B–D) AAV‐AQP4 or AAV‐eGFP was treated to NOTCH3‐R170C mice (16 weeks of age). Coronal brain sections were subjected to immunostaining of indicated markers. (B) Area of P16 or P21 coverage. *N* = 6 in each group. ***p < 0.001; by one‐way ANOVA (mean ± SEM). (C) Quantification of the percentage of P16^+^ cells among neurons (NEUN^+^), oligodendrocyte (CC1^+^), microglia (IBA1^+^), or astrocyte (S100B^+^). *N* = 6 in each group. *p < 0.05, **p < 0.01; by one‐way ANOVA. Difference of means (± SEM) is displayed. (D) Quantification of the percentage of P21^+^ cells among neurons (NEUN^+^), oligodendrocyte (CC1^+^), microglia (IBA1^+^), or astrocyte (S100B^+^). *N* = 6 in each group. *p < 0.05, **p < 0.01; by one‐way ANOVA (mean ± SEM).

We further analyzed the therapeutic effect of AAV‐AQP4 treatment against brain senescence. Performing senescence‐associated β galactosidase (SA‐β‐GAL) staining, we found that NOTCH3‐R170C mice displayed advanced brain aging, which as early as 16 weeks of age (Figure 6A). Consistently, expression of the senescence markers P16 and P21 was integrally increased among brain cells in the CADASIL models (age = 16 weeks) (Figure 6B), which was most prominent in neurons (NEUN^+^) (Figure 6C,D). Moreover, at later stage of age (age = 48 weeks), the CADASIL models displayed increased neuronal death (Figure S10A) and severe white matter injury (Figure S10B). As AQP4 expression was reinforced, the levels of P16 and P21 were downregulated in the AAV‐AQP4‐treated NOTCH3‐R170C mice when compared with the AAV‐eGFP‐treated controls (Figure 6B–D). In comparison, WT mice that received AAV‐AQP4 treatment did not show further decreased expression of senescence markers (Figure S11D,E). The data illustrate that when AQP4 expression and glymphatic function are intact, to enhance AQP4 expression could neither improve glymphatic activities nor affect brain cell senescence. However, NOTCH3 mutation leads to decreased AQP4 expression and glymphatic impairment. In this case, to reinforce AQP4 expression resumes glymphatic functions and ameliorates brain cell aging in CADASIL.

## 4 Discussion

The current study demonstrates that glymphatic functions are impaired in CADASIL. We show that NOTCH3 mutation results in hypo‐activation of RUNX1‐CMYB signaling, thus reducing AQP4 transcription in astrocytes. Reinforcing AQP4 expression resumes glymphatic activity and prevents subsequent brain senescence. We thus purpose that glymphatic system is a potential therapeutic target in CADASIL.

As a critical molecule of glymphatic system, expression of AQP4 requires subtle regulation. In the current study, we show that NOTCH3‐RUNX1‐CMYB is among the pathways that promote *Aqp4* gene expression. However, NOTCH3 is implicated in the complex cellular signaling network. Functions of NOTCH3 could affect and be affected by other factors including NOTCH1 and NOTCH2. As far as we are concerned, NOTCH3 is “one of”, instead of “the only” regulating hub of AQP4 expression. Concurrently, the NOTCH3‐RUNX1‐CMYB signaling represents one of the regulating pathways that mediates AQP4 expression, other responsive signaling that are also involved remained to be revealed.

NOTCH3 mediates cell proliferation, differentiation as well as apoptosis [58]. NOTCH‐RUNX1‐CMYB pathway is involved in critical biologic processes including the regulation of stem cell development [4, 11]. Furthermore, we recorded that downregulation of NOTCH3‐RUNX1‐CMYB pathway in NOTCH3‐mutant CADASIL models resulted in diminished AQP4 expression, which led to glymphatic dysfunction. Since the glymphatic system is responsible for metabolic waste drainage, glymphatic failure in CADASIL may lead to accumulation of metabolic waste in the brain, which promote brain cell senescence. Therefore, we infer that hypo‐activity of NOTCH3‐RUNX1‐CMYB‐AQP4 pathway promotes brain cell senescence through attenuation of glymphatic function.

Previous report revealed that astrocytic endfeet were retracted in white matter lesion of CADASIL patients. As a result, AQP4 was preferentially localized in the cell body [59]. According to our results, reduced AQP4 expression is evident in CADASIL mouse model as early as 16 weeks of age. Nevertheless, abnormal AQP4 distribution along blood vessel were not evident until 24 weeks. We thus infer that at early stage of CADASIL, it is diminished AQP4 protein level rather than impaired AQP4 polarization that contributes to the retarded glymphatic functions.

Our data illustrate that AQP4 inhibitor would not directly cause brain cell senescence. To be noticed, glymphatic system conducts clearance of metabolic waste and abnormal protein in CNS [57, 60, 61]. According to previous reports, AQP4 deficiency decreases drainage of amyloid protein beta and Tau [62, 63, 64], which are potent pro‐senescent factors. Nowadays, detrimental impacts of glymphatic failure to brain senescence have attract increasing attention [17, 65, 66]. As far as we are concerned, glymphatic dysfunction causes accumulation of pro‐aging metabolic waste and abnormal proteins, which subsequently accelerates brain cell senescence. The current study demonstrates that NOTCH3 mutation leads to reduced AQP4 expression and impaired glymphatic functions. At the meantime, CADASIL brains display advanced senescence. Reinforcing AQP4 expression in astrocytes not only resumes glymphatic functions but also prevents advanced brain senility in CADASIL mice, which highlights the potential of glymphatic system as a therapeutic target in CADASIL. Accumulation of myelin debris due to white matter injury and deposition of GOM are both pathologic signatures of CADASIL. The impacts of AQP4 reinforcement on the burden of myelin debris and GOM in brain remain to be studied. On the other hand, cerebral ischemia is commonly manifested in CADASIL patients [1, 67] which provokes brain tissue edema [68, 69]. In acute ischemic stroke, excessive AQP4 function exacerbates brain edema, leading to neuronal loss and deteriorated prognosis [70, 71]. The possible side effects of the treatment in CADASIL patients with ischemic attack should be seriously taken into consideration.

Recent report has revealed that inactivation of *Notch3* accelerated VSMC aging with progressive dedifferentiation and detachment of VSMCs, leading to the structural abnormalities and deficiencies in contractility of blood vessel. Furthermore, the absence of *Notch3* has been linked to a dysfunction in the glymphatic system, manifesting as an increased accumulation of chondroitin sulfate‐positive material within the brain parenchyma, which ultimately contributes to neurodegeneration [21]. Our data illustrated that in addition to the impact of smooth muscle cells and pericytes on glymphatic function, *Notch3* mutation directly leads to the downregulation of AQP4 expression in astrocytes, which in turn, exacerbates glymphatic dysfunction. We also noticed that *Notch3* was expressed in microglia. Recent reports have revealed that using PLX5622 to deplete microglia or P2Y12 knockout mice showed enhancement of glymphatic system function. Additionally, chemogenetic activation of microglia enhanced glymphatic system function [72, 73]. We hypothesize that microglia may be involved in glymphatic function. However, the role of microglia in glymphatic system and the impact of microglia in CADASIL needs to be further investigated.

Immunotherapy targeting extracellular domain (ECD) of NOTCH3 enhances the clearance of GOM, thus ameliorates the toxicity of the deposits and protects against cerebral vascular injury [2, 74]. Instead of focusing on the existed pathologic GOM, the current study unveils the functional deficit of glymphatic system which is directly caused by NOTCH3 mutation and proposes a corresponding gene therapy. In Alzheimer's disease models, it has been found that to enhance lymphatic drainage improve the therapeutic effects of anti‐amyloid immunotherapy [75]. We thus speculate that enhancement of glymphatic function combined with immunotherapies could lead to better therapeutic effects in CADASIL.

In conclusion, the current study elucidates the impairment of glymphatic functions in CADASIL. AQP4 expression in astrocyte is diminished due to the hypo‐activation of NOTCH3‐RUNX1‐CMYB signaling. AQP4‐reinforcing therapy resumes glymphatic functions and thus prevents brain senescence. However, application scope and potential side effects of the therapy should be considered with deliberation.

## Author Contributions

C.L. designed and performed the experiments, collected and analyzed the data, and drafted the manuscript. H.L. and XM contributed to the experimental design and revised the manuscript. X.K. and Y.W. contributed to the experimental design and the manuscript. M.H. and X.S. collected the clinical samples. S.W., W.C. collected the clinical data and revised the manuscript. D.L., S.S., and L.Z. performed the animal experiments and collected the data. H.H., Y.L., and X.D. collected the clinical data and revised the manuscript. A.W. and Z.L. designed and supervised the study and critically revised the manuscript. The authors read and approved the final manuscript.

## Ethics Statement

All animal experiments were approved by the Third Affiliated Hospital of Sun Yat‐sen University and performed following the Guide for the Care and Use of Laboratory Animals and Stroke Treatment. Clinic research was approved by the ethics committee of the Third Affiliated Hospital of Sun Yat‐sen University.

## Consent

The authors have nothing to report.

## Conflicts of Interest

The authors declare no conflicts of interest.

## Supporting information


Data S1.

## Data Availability

Mouse single‐cell mRNA sequencing data generated for this study can be found in the GEO repository under accession number GSE257560. Further information of this study is available upon reasonable request from the corresponding authors. The public datasets used in this study include the following: Ximerakis et al. [48] (GEO accession number: GSE129788), Wei et al. [49] (GEO accession number: GSE211099), Depp et al. [50] (GEO accession number: GSE178304), and Wheeler et al. [51] (GEO accession number: GSE120119). All other data needed to evaluate the conclusions in the paper are present in the paper and/or the Supporting Information.
