# Supplementary material for: NOTCH3 Mutation Causes Glymphatic Impairment and Promotes Brain Senescence in CADASIL
Source: CNS Neurosci Ther. 2025 Jan 24;31(1):e70140. doi: 10.1111/cns.70140 (PMC11760990; doi:10.1111/cns.70140)
Supplement: Supplementary file 1 — Data S1. [file CNS-31-e70140-s001.docx]

**Supplementary figures**


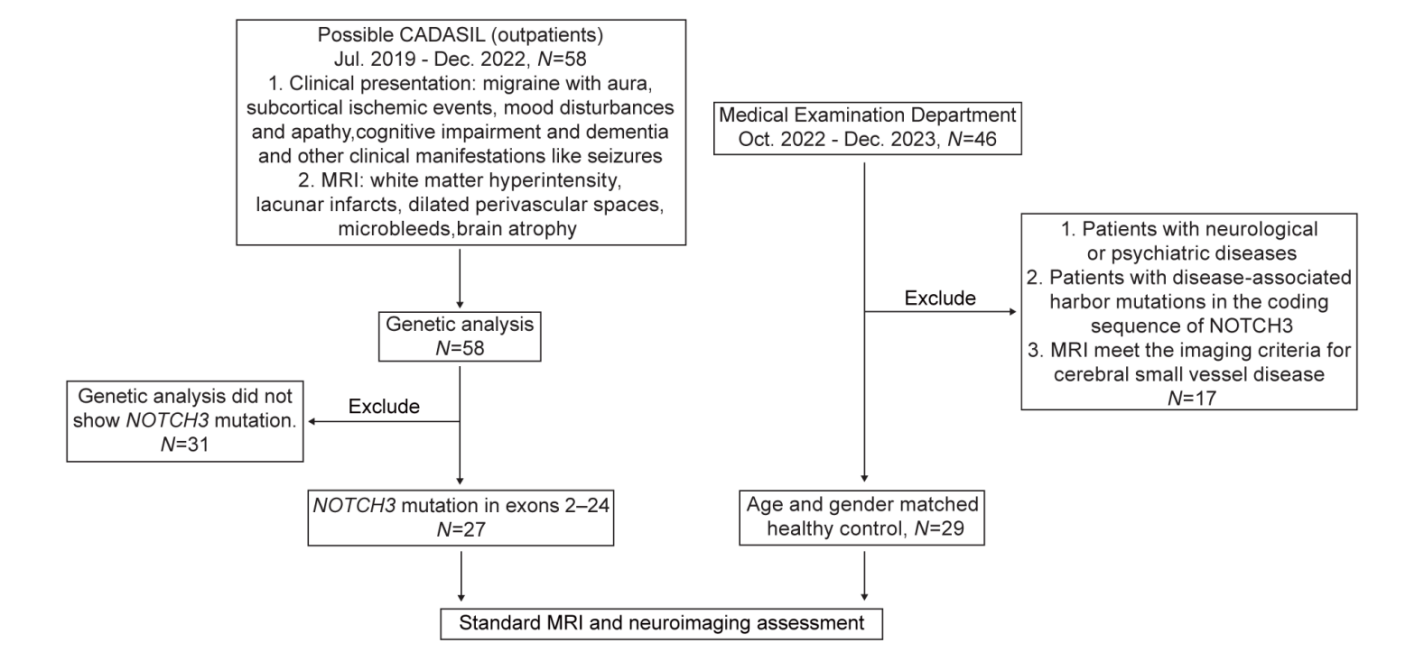


Fig. S1. Inclusion process of CADASIL patients and healthy controls in the recruited cohort.


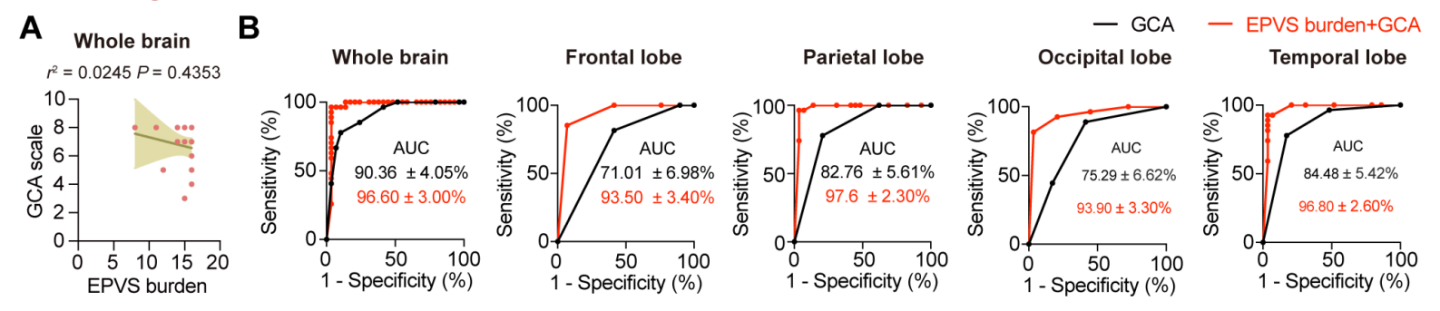


Fig. S2. Inclusion of EPVS burden improves the diagnosis-predicting efficacy for CADASIL of GCA scale. (A) Correlation of EPVS burden and GCA scale in the whole brain of CADASIL patients (*N* = 27) was analyzed with simple linear regression. (B) Prediction efficacy of GCA scale with or without EPVS burden for CADASIL diagnosis (True = CADASIL, False = HC) was assessed with ROC analysis. *N* = 29 in HC and *N* = 27 in CADASIL.


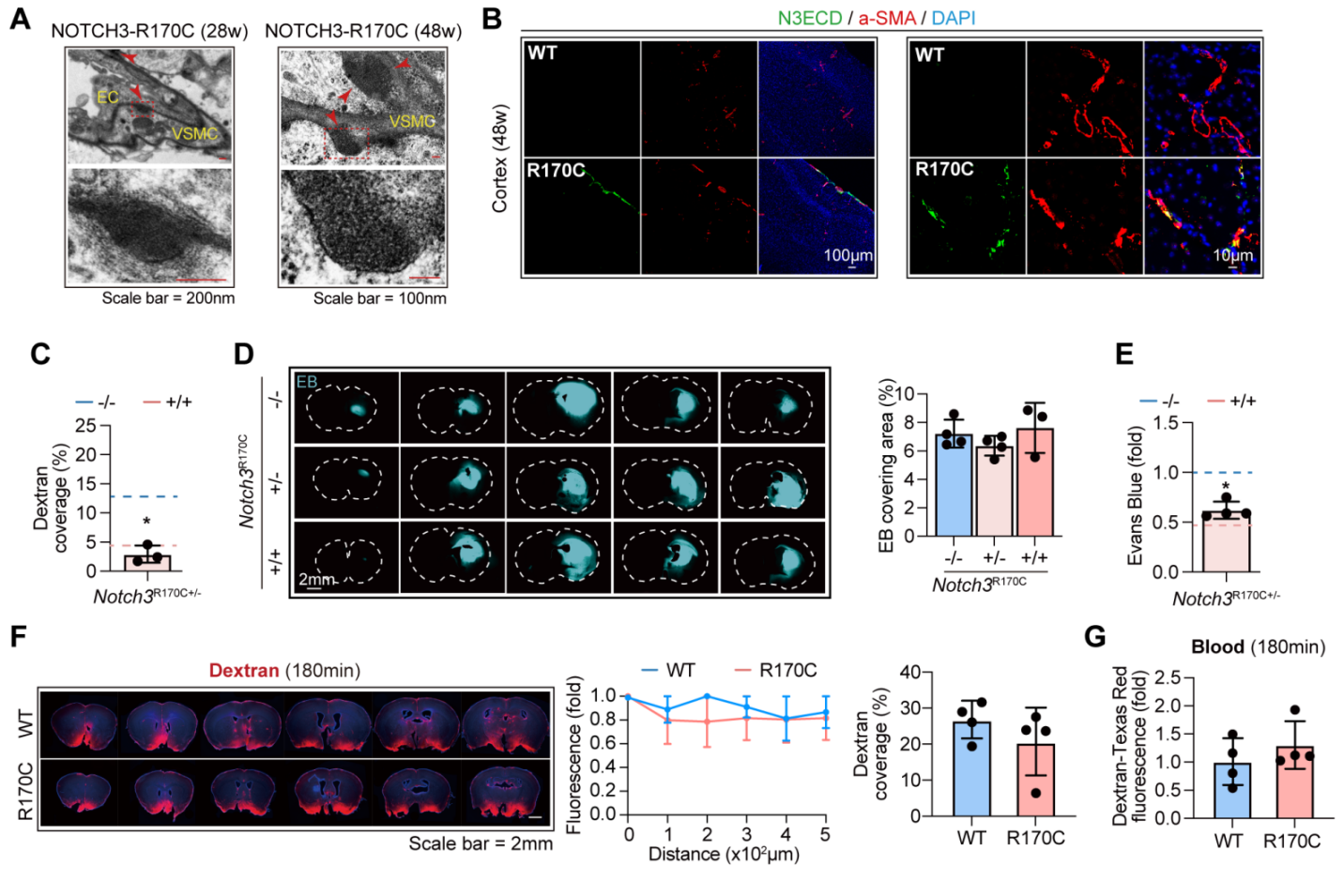


Fig. S3. Evaluation of glymphatic activities in NOTCH3-R170C models. (A) Brain tissue of NOTCH3-R170C mice (age = 28w and age = 48w) was sent to TEM. Representative images showing the deposition of granular osmiophilic material (GOM) are displayed. (B) Coronal brain sections of NOTCH3-R170C or -WT mice (48w of age) were subjected to immunostaining of N3ECD (green), а-SMA (red) and DAPI (blue). (C) Dextran coverage in heterozygous *Notch3*^R170C+/-^ mice (16w of age) at 30min after i.cm. injection. *N* = 3. **P* < 0.05; compared with WT, by one-way ANOVA (mean ± SEM). (D) Representative images for EB covering area at 90min after injection into the striatum of heterozygous *Notch3*^R170C+/-^  mice (16w of age). EB infusion was calculated in the indicated groups. *N* = 4 in *Notch3*^R170C-/-^  (NOTCH3-WT) and *Notch3*^R170C+/-^  groups; *N* = 3 in *Notch3*^R170C+/+^  (NOTCH3-R170C) group. Statistics analysis was performed with one-way ANOVA. (E) EB concentration in blood of heterozygous *Notch3*^R170C+/-^ mice (16w of age) at 90min after injection. Data ware showed as fold change to NOTCH3-WT mice. *N* = 4. **P* < 0.05; by one-way ANOVA (mean ± SEM). (F) Fluorescence intensity of Dextran at indicated distance from brain surface and Dextran coverage in NOTCH3-R170C and -WT mice at 180min after i.cm. injection. *N* = 4 in each group. Statistics analysis was performed with one-way ANOVA (mean ± SEM) or Student’s *t* test (mean ± SEM). (G) Fluorescence intensity of Dextran at blood in NOTCH3-R170C and -WT mice at 180min after i.cm. *N* = 4 in each group. Statistics analysis was performed with Student’s *t* test (mean ± SEM).


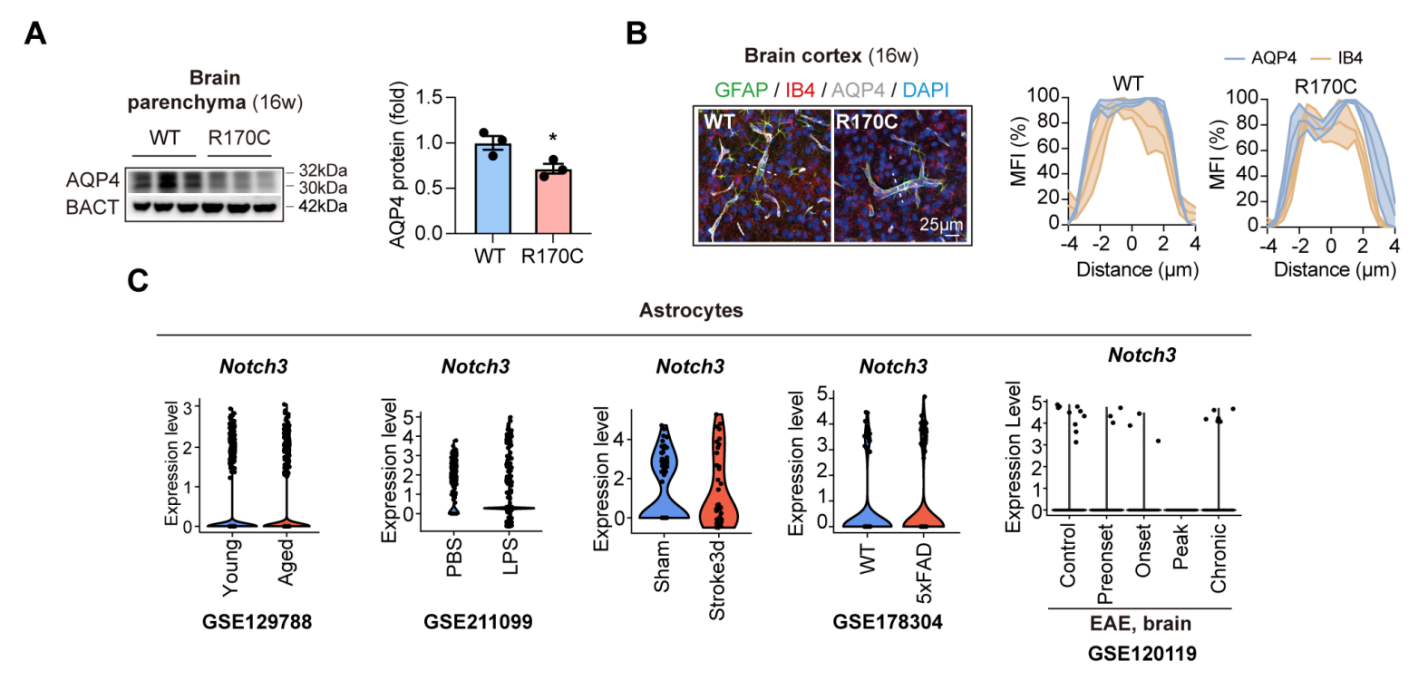


**Fig. S4. AQP4 expression and distribution in 16w-old NOTCH3-R170C mice.** (**A**) Brain protein of NOTCH3-R170C or -WT mice (16w of age) was subjected to western blot to evaluate AQP4 expression. *N* = 3 in each group. **P* < 0.05; by Student’s *t* test (mean ± SEM). (**B**) Coronal brain sections of NOTCH3-R170C or -WT mice (16w of age) were subjected to immunostaining of GFAP (green), IB4 (red) and AQP4 (gray). AQP4 distribution centered on blood vessels (IB4^+^) was evaluated. (**C**) Violin plots of *Notch3* expression in astrocytes at various scRNAseq data of neurological diseases, including aging, LPS-induced inflammation, stroke, Alzheimer disease and experimental autoimmune encephalomyelitis (EAE).


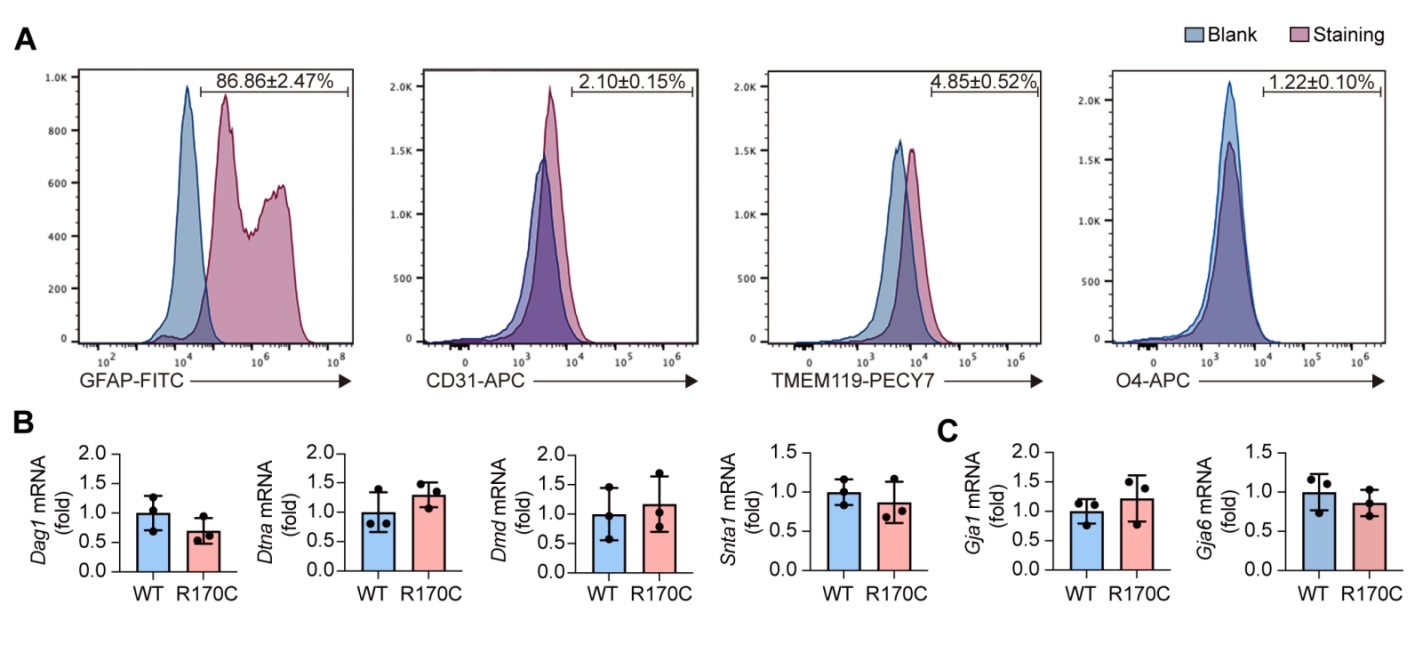


**Fig. S5. Transcription of dystrophin-associated complex and gap junction associated genes in NOTCH3-R170C astrocyte.** (**A**) Purity of the primary astrocyte enriched cultures were assessed with flow cytometry. Proportion of astrocytes (GFAP^+^), blood vessel endothelial cells (CD31^+^), microglia (Tmem119^+^) and oligodendrocytes (O4^+^) in the cultures was calculated. Experiments were repeated for 3 times. Difference of means (± SEM) is displayed. (**B-C**) Primary cultures of NOTCH3-R170C or -WT astrocytes were subjected to RT-PCR to analyze the transcription of dystrophin-associated complex (DAC) (**B**) and gap junction (**C**) associated genes. Data were interpreted as fold change to NOTCH3-WT group after normalization to *Actb* mRNA level. Experiments were repeated for 3 times. Statistics analysis was performed with Student’s *t* test.


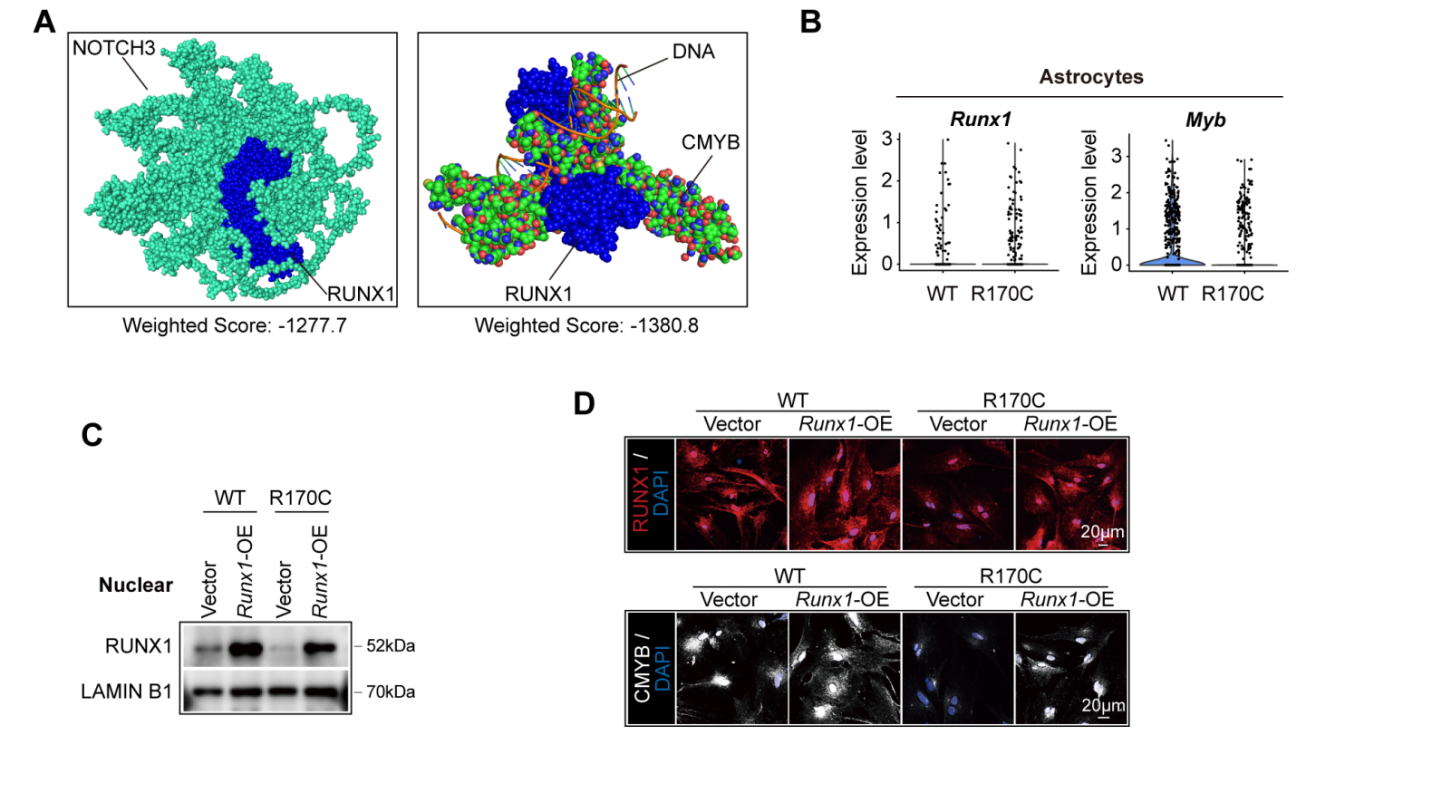


**Fig. S6. RUNX1 overexpression reinforces RUNX1 activation in NOTCH3-R170C astrocytes.** (**A**) Molecular docking showed the interaction between NOTCH3 and RUNX1 or RUNX1 and CMYB. The weighted score were obtained from Cluspro 2.0 docking server and images were performed by PyMOL. (**B**) Brain cells of NOTCH3-R170C and -WT mice (16w of age) were subjected to single cell RNA sequencing (scRNAseq). Violin plots of *Runx1* and *Myb* expression in astrocytes are displayed. (**C-D**) RUNX1 was over-expressed in NOTCH3-R170C or -WT astrocytes with lentiviral vectors carrying *Runx1* cDNA (*Runx1*-OE). (C) Nucleus protein of indicated groups were subjected to western blot. (**D**) The *Runx1*-OE astrocytes were subjected to immunostaining of RUNX1 (red) and CMYB (gray).


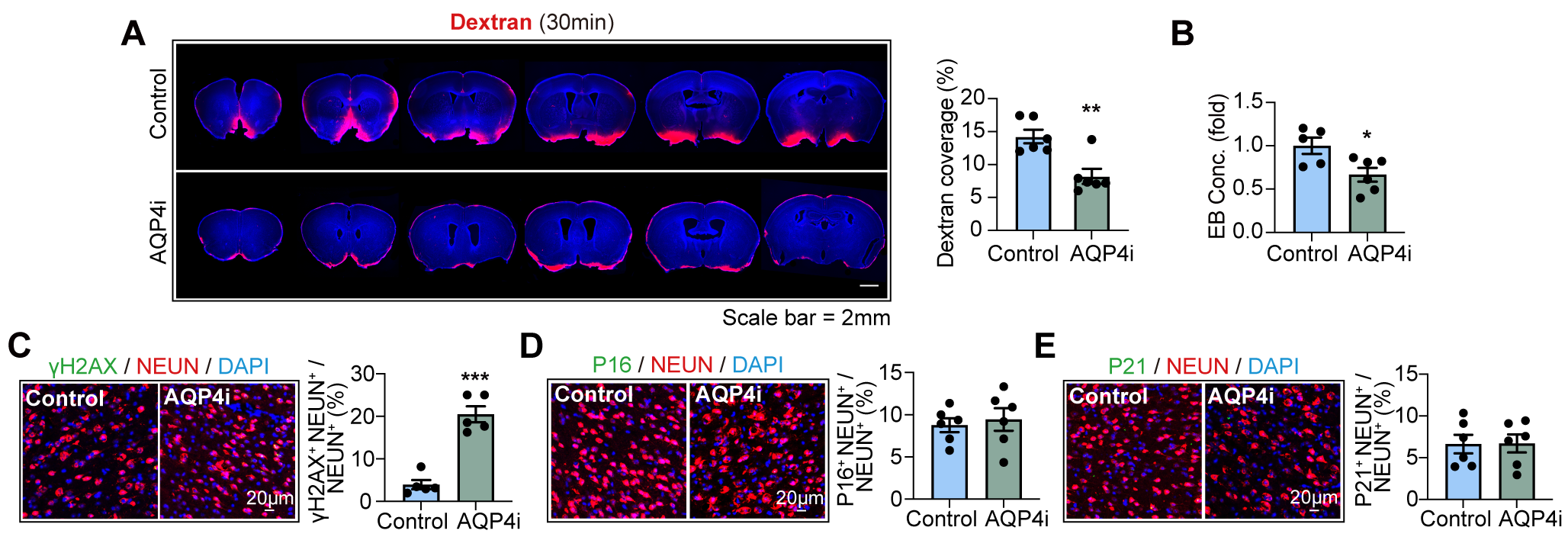


**Fig. S7. Evaluation of glymphatic efflux function and brain senescence in AQP4i models.** AQP4 inhibitor TGN-020 (2.5μg in 5μl PBS, i.cm, twice a week) or PBS was administrated to WT mice (16w of age) . Mice were sacrifice at 7d after the first injection. (**A**) The fluorescent tracer of 3kDa Dextran-Texas Red was injected into the CSF (i.cm., 10mg/ml in 5μl) to evaluate glymphatic influx. At 30min after injection, mice were sacrificed and the coronal brain sections were subjected to microscopy. Dextran coverage in the whole brain was analyzed. *N* = 6 in each group. ***P* < 0.01; by Student’s *t* test (mean ± SEM). (**B**) A total of 1μl EB (960Da, 40mg/ml) was injected into striatum and blood EB level at 90min after injection was assessed to evaluate glymphatic efflux. *N* = 5 in control gourp and *N* = 6 in AQP4i-treated group. **P* < 0.05; by Student’s *t* test (mean ± SEM). (**C**) Quantification of the percentage of γH2AX^+^ NEUN^+^ cells among NEUN^+^ neurons and representative images. *N* = 5 in each group. ****P* < 0.001; by Student’s *t* test. Difference of means (± SEM) is displayed. (**D-E**) Quantification of the percentage of P16^+^ NEUN^+^ cells (**D**) and P21^+^ NEUN^+^ cells (**E**) among NEUN^+^ neurons and representative images. *N* = 6 in each group. Difference of means (± SEM) is displayed.


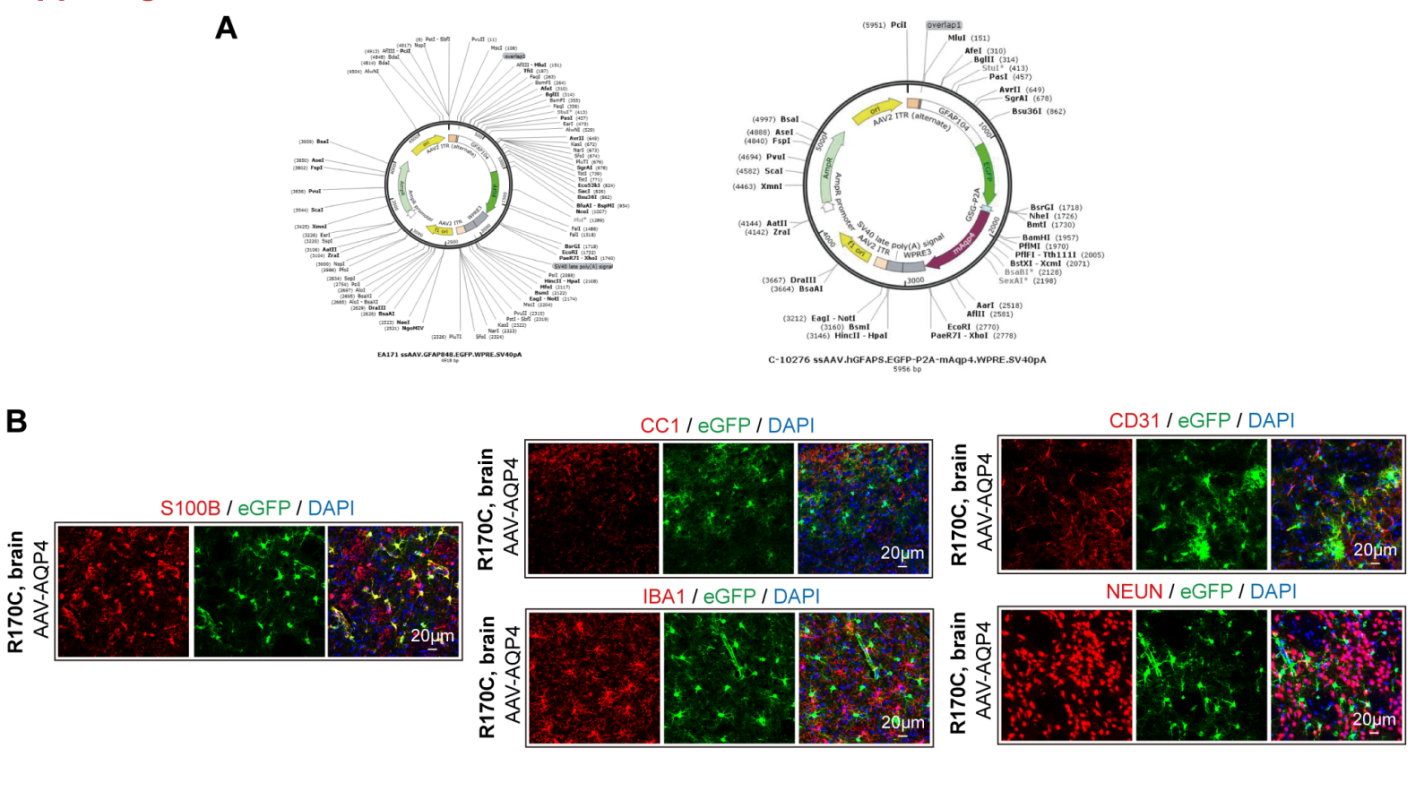


**Fig. S8. Atlas of AAV-eGFP and AAV-AQP4.** (**A**) EGFP and *mAqp4* is separated with GCG-P2A, which facilitate the dissociation of the translational products of AQP4 and eGFP. (**B**) AAV-AQP4 (ssAAV.hGFAPS.EGFP-P2A-AQP4-M1) or AAV-eGFP (ssAAV.hGFAPS.eGFP) was treated to NOTCH3-R170C mice at the age of 16w (Stereotactic injection into striatum). Mice were sacrificed at 21d after injection. Coronal brain sections of mice were subjected to S100B, CC1, IBA1, CD31 and NEUN (red) staining. Green fluorescence of eGFP was recorded simultaneously. Representative images are displayed.


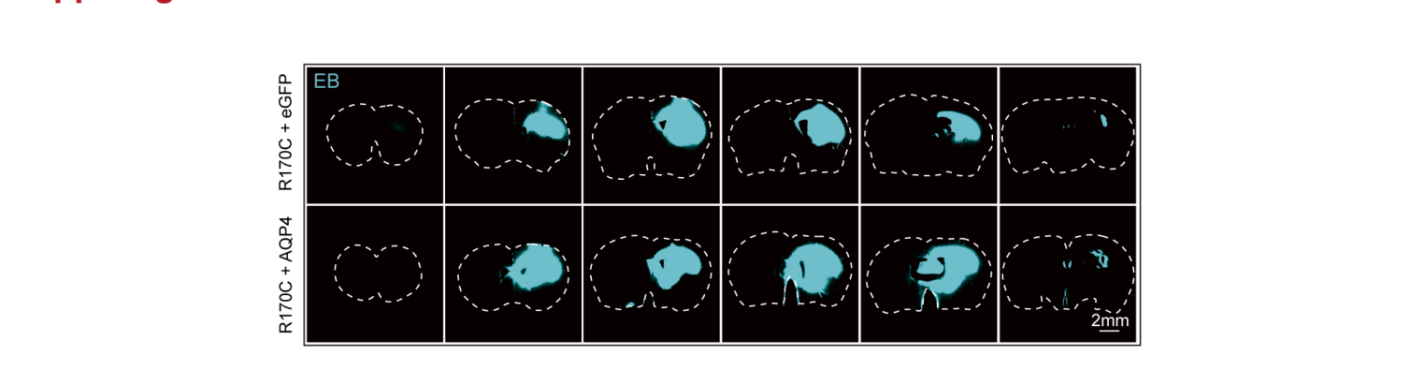


**Fig. S9. Stability of EB infusion volumes in glymphatic efflux analysis of AAV-treated CADASIL mice.** Representative images for EB covering area at 90min after injection in indicated groups.


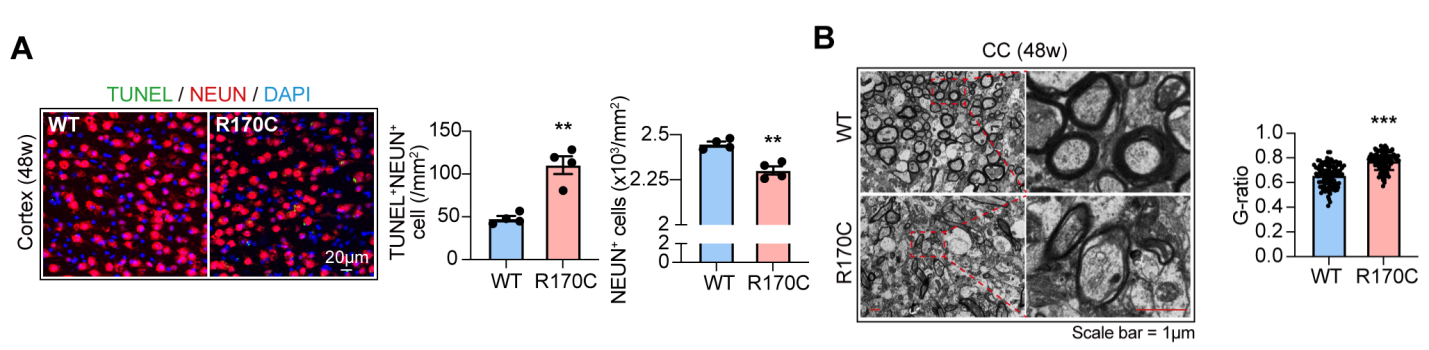


**Fig. S10. Neuronal death and white matter injury in NOTCH3-R170C mice.** (**A**) Coronal brain sections of NOTCH3-WT and NOTCH3-R170C mice (48w of age) were subjected to TUNEL (green) and NEUN (red) staining. *N* = 4 in each group. ***P*<0.01, by Student’s *t* test. (**B**) Brain tissue of NOTCH3-WT (3 mice) and NOTCH3-R170C mice (3 mice) (48w of age) was subjected to transmission electron microscopy (TEM). G-ratio (axon diameter / myelin thickness) was computed to evaluate white matter injury, with at least 100 axons being analyzed per animal. ****P*<0.001, by Student’s *t* test. Difference of means (± SEM) is displayed.


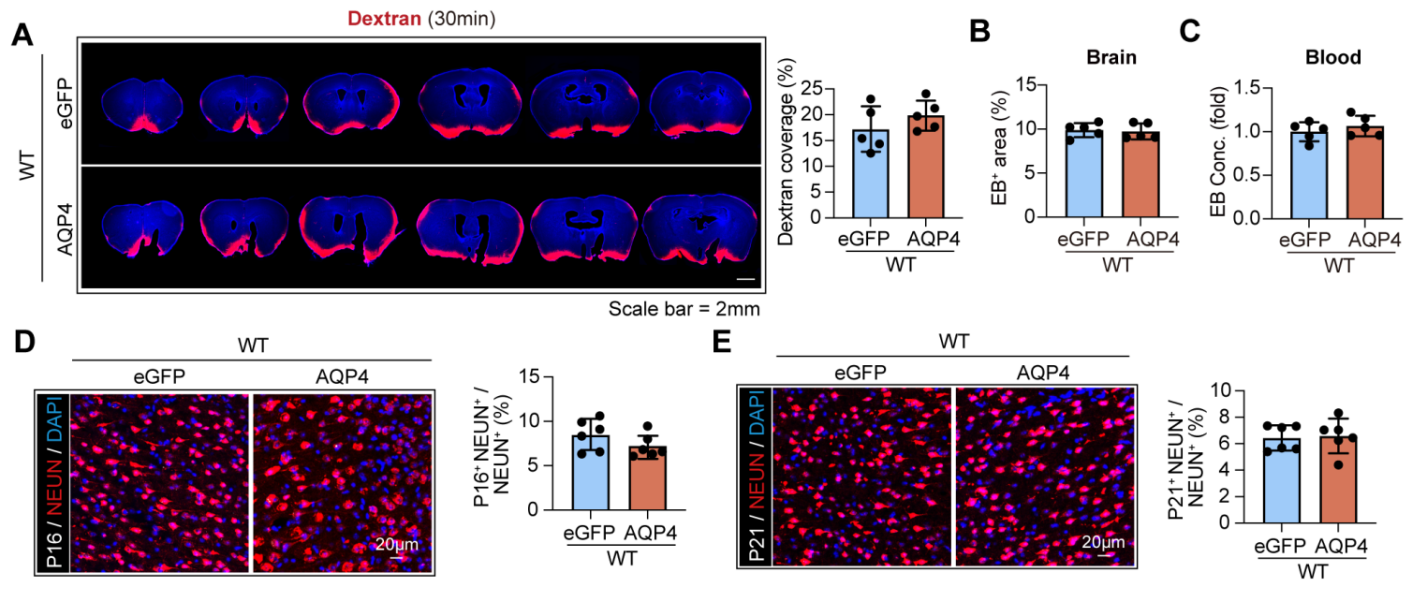


Fig. S11. Evaluation of glymphatic activities and brain senescence in WT mice of AAV-based gene therapy. AAV-AQP4 (ssAAV.hGFAPS.EGFP-P2A-AQP4-M1) or AAV-eGFP (ssAAV.hGFAPS.eGFP) was treated to NOTCH3-WT mice at the age of 16w (Stereotactic injection into striatum). Mice were sacrificed at 21d after injection for analysis of glymphatic activities (A-C) and brain senescence (D-E). (A) The fluorescent tracer of 3kDa Dextran-Texas Red was injected into the CSF (i.cm., 10mg/ml in 5μl) to evaluate glymphatic influx. At 30min after injection, mice were sacrificed and the coronal brain sections were subjected to microscopy. Dextran coverage in the whole brain was analyzed. *N* = 5 in each group. By Student’s *t* test (mean ± SEM). (B-C) To evaluate the function of glymphatic efflux, a total of 1μl Evans blue (EB) (960Da, 40mg/ml) was injected into striatum and blood EB concentration at 90min after injection was assessed. (B) EB covering area in brain. *N* = 5 in each group. (C) EB level in plasma. *N* = 5 in each group. (D-E) Quantification of the percentage of P16^+^ NEUN^+^ (D) or P21^+^ NEUN^+^ (E) cells among NEUN^+^ neurons in AAV-AQP4 or AAV-eGFP treated NOTCH3-WT mice. Difference of means (± SEM) is displayed.

Supplemental Tables

**Table 1. Clinical characteristics of the individuals involved in the study.**

| **Clinical Characteristics** | **Healthy controls** | **CADASIL** |
| --- | --- | --- |
| *N* | 29 | 27 |
| Age, y | 51.79 ± 9.12 | 49.67 ± 12.58 |
| Male, n (%) | 14 (48.28%) | 12 (44.44%) |
| Female, n (%) | 15 (51.72%) | 15 (55.56%) |

**Table 2. Primers used in the study.**

| **Gene** | **Forward primer** | **Reverse primer** |
| --- | --- | --- |
| *Actb* | AGGGAAATCGTGCGTGACAT | GAACCGCTCATTGCCGATAG |
| *Dag1* | GAGCAGTGAGGACGATGTTTA | CCCTTCCTCTTCTTGCGATAG |
| *Dmd* | GCTTCTAGTCATCTGGGCTTATC | ACATCTGGAGCCCTAGACAATA |
| *Dtna* | GTCCACTTCACACCTCTTTAGTT | GAAAGGTCCTCAGGAAGAATGG |
| *Snta1* | GATTGGCTGGCTGACAGAA | CTGAGGGAGAGAGCAGTAGAA |
| *Gja1* | GGTGTCTCTCGCTCTGAATATC | GTAAGGATCGCTTCTTCCCTTC |
| *Gja6* | CCGCCTTCATGTATGTGTTCTA | TGAAGCAGTCCACGAGATTG |
| *Notch3* | CGTGTGGCCTCTTTCTACTGT | GCACCAATCGAGCACTCATC |
| *Aqp4* | ATCAGCATCGCTAAGTCCGTC | GAGGTGTGACCAGGTAGAGGA |

**Sequence information**

The mAqp4 coding sequence was GCCACATGGTGCAGAATCTTTCCACCCCTACTCTCCAAAAACCCAATCAGACAAGTGCCCGTAATCTGACTCCCAGTGTACTGGAGCCCGGGGGCAGGCACTGAGCTGCACTCTGGCCAGGGAAGGCATGAGTGACAGAGCTGCGGCAAGGCGGTGGGGTAAGTGTGGACATTCCTGCAGTAGAGAGAGCATCATGGTGGCTTTCAAAGGAGTCTGGACTCAGGCTTTCTGGAAGGCAGTCTCAGCAGAATTTCTGGCCACGCTTATCTTTGTTTTGCTCGGTGTGGGATCCACCATAAACTGGGGTGGCTCAGAAAACCCCTTACCTGTGGACATGGTCCTCATCTCCCTTTGCTTTGGACTCAGCATTGCTACCATGGTGCAGTGCTTTGGCCACATCAGTGGTGGCCACATCAATCCCGCTGTGACTGTAGCCATGGTGTGCACACGAAAGATCAGCATCGCTAAGTCCGTCTTCTACATCATTGCACAGTGCCTGGGGGCCATCATTGGAGCCGGCATCCTCTACCTGGTCACACCTCCCAGTGTGGTTGGAGGATTGGGAGTCACCACGGTTCATGGAAACCTCACCGCTGGCCATGGGCTCCTGGTGGAGTTAATAATCACTTTCCAGTTGGTGTTCACTATTTTTGCCAGCTGTGATTCCAAACGAACTGATGTTACTGGTTCAATAGCTTTAGCAATTGGATTTTCCGTTGCAATTGGACATTTGTTTGCAATCAATTATACTGGAGCCAGCATGAATCCAGCTCGATCTTTTGGACCCGCAGTTATCATGGGAAACTGGGCAAACCACTGGATATATTGGGTTGGACCAATCATGGGCGCTGTGCTGGCAGGTGCCCTTTATGAGTATGTCTTCTGTCCTGATGTGGAGCTCAAACGTCGCCTTAAGGAAGCCTTCAGCAAAGCCGCGCAGCAGACAAAAGGGAGCTACATGGAGGTGGAGGACAACCGGAGCCAAGTGGAGACGGAAGACTTGATCCTGAAGCCCGGAGTGGTGCATGTGATTGACATTGACCGTGGAGAAGAGAAGAAGGGGAAAGACTCTTCGGGAGAGGTATTGTCTTCCGTATGACTAGAGGACAGCACTGAAGGCAGAAGAGACTCCCTAGACCTGGCCTCAGATTTCCTGCCACCCATTAAGGAAACAGATTTGTTATAAATTAGACACTTGCGGGTTTCTTGCTTCACACCTTGTTACACAGTTTAAATAACACATATTTTACTATTATCAATGGGGGGGGTGAGAAAAAGCCTATAATGGATATAAAATTTTAAAACAGAAATCTTTTTGAATGTATCCCCAAAGCAACTATGCAACTAGTGTATTGGCTTCCCTTTCATTAATAACTGAAATTATGAACCAAGATCTGGTCAAGTTTTGCCGTGCAGAGCATATGGACACCTCTGTGAGGAAGCTGGCATTGTCCATCGTCTTGACTATTGACTTCATTGGATTGATTTAAAAATGACAAAATGCAGTATGTCACAGAATCATGTGCATTCAGGAGAAGACATGCAGTAACTTCTTCCACGAGCTATTCCTTATTTATAAACTACCTCGGAGGGGGAAAACATTAGCAAGGGCCATTGCTAATACATCATTTGTATTTATATATCTGACTGTCAGAACCTAACTCTAATTCACTATAATCCTCTCCCCCTCAGAATCCAAGAACCCATAGGGCTAAAAGTAAAAAAAAAATAGGAAAAAATATTAATTTCTGTCCATGTGTTAGTTCCATCAAATGAAAGCTATACATGGGACACCCATTGAGGTTCAGGACAGCCAGACTTGGGAGTCTGGTGGGAGGTGGAACAAGGTGTGCTACTGCCTTGCATCCATCACACGGAGAGGGAAACCTCTGTTATCAAATGATTGGTCTGTTTTCAGTGCACACTCTTAAATGTACCACAAACCATTAACCACCTAAGCTTTTAACTATTTCACTGACTTTTTAAAGCTTATTTGCAAAACTGGGGAATTTGTTTGCCATTTTGATCCCTAAATACTAGCAGAGACATTTTAGACCAGAACTTGACTAGTCGAGTCCTGACTTTTAGTTGGTGTCTAAATCCTGCTTACTCTAGCTGGTATAAACCAGTCCTGCCCCATCTTAGCTGCTGATGCTGTTTTGATTCCCACGATATGCATCGACAATCGGCATTGTGAGTGTAATTATACCCATTTGTGTGAATTAAAATATATGCAGACAAGGTGCAACGTGGTTGCCAAATAAAGGGGTGAGCACACAGCCTCTGTGCAAAGCTCCTAGTTACCCCATCTGCACATAGCTTACCATCATGCTTTTAAGGTATTTACCTCCTGCTTGGTTTATTTGTTAAAACCATTTATTTCTCCCATACTGCTTTGCCTTCCGCCCATCGAATGCTCTGTGGAAACCCCAGTTTCTATGATCATAACAGTCTAGCATAGCATTTATCTTAGACAATGTGCTGCAAATGTCGATTTCAGTAAACAAAAGTTAGTAGTGGAAGAGAGAAGAGCCATTTCTTCAAGGACTAGCTTGTAAATAGCTGGTAATGGAGGGCTTTCTTCTCCCAAGCACAATTCCAACTGTGTGTGACTTCTTATTGTTAATGGCAGTTTTGTGTCTGTGGCAGCGAGATAATGGACCACTATTAAACCTGATTCTCTTCGGTGCTAGGAAACTGATGTGTGAGTTCCCAGAAAGGCACGAGGGCAGCATATGCCTCTCGGTGCCATGGTCATGTTCTGAGGCAGATGCTGGGAGCATGGGCCACATTTCAGGGCTTTGAATATACTACACCAGAGACATAAGCTTCCTCTCTGAGCAGATCACGCAGAACCAGGGCATAGACCTCCCAGGGGAGGTTTCTGCCGATTCTTTCAAATGTTTTCACAATATACCACGCAGACTTAAGATCAGAGCGCTTCAGTTCGGAAGGTGATATCAACCATCAACAAATATGTCGGGTCAAAAATAAATTTACTTAACAAGGAATAGTTAAGCACATTTCTGATTACTCTTTCAAAATCCTGGCCTGTGATTATTTTTATTATGCTAGACAGCTTGCTTCGTTTGTGCTCACTTTAACAGTCATTTCCAGTGACAGCATTCAGTATGGTACAAACTGAAAAACAGGGAATCATAGCTTAGTGATTTGTTTGCTAGCAGCTGGCAGAGTGCCAGGCACACAGTAGGCAATCAAAACAAGCATGTTGTTTGAATATATATACATATATACATATATACATATATACATATATACATATATGTGTATATATGATTCTTATTTGGGATTTAAAATACCAATAATCTCCTCTAATTTTTAATTTTGCCCAATATTTAGTGAAAACATAATTTTTAGTGTCATCAATAAAAGCAACATGGACTTGGAAACAAAGAGCATATTTTTACATTCTACTGTTTGAAGGCAGAGAGTGACTACTTCACACTCTTCGTCTTTGCAATATGTCTTGCATTTCACTCACGGCTCTGCCAGTAAAAAATGTAATGAAATTGTCCCTTTCTAATGACATCGATGCAGCAGGGGTCTATTGCCTTGTGGATGACACCAAATTATACAACATATTAGGAGATCGGGGATTTTCCCTTTAATCCCTTCTTATTAATGAAGTGCATAGTGCCGTTCCCAAGAGACAGCTACTGACAGATACACCGCACAGAGATCAGAGAGGAAAAATCAGGAAGACATAAAAGATTTATACGAGCCATGAAACAATGCCAACTGTCTGTTCCCTCAGGAGGAGAACACAGACACACCACAAATTTCAAGGTGGTCCATACACTGGAAATGTACATACATAGTCTGTCAAAGGAGAACAGAAGGAAATCTTTTTTTTTTAATTTTGAGCATTTTTTTTTTCAAATCAGAGACTCTGATTTTTAAATGTGTGTTTCCATTTTCTTCCAAATGTTCTACCCTTTTATACTACAAGAAACTGTTTCCTGTAGCCTAAGTCACTGGTAATTTTACAACTTGTTCCTGTGATTTGCCACTATCATGAGTCCTCTTCCGTTCGATCTTCAGAGGGGTGTCCCACCACAGCTCGGACACTGCTGGCCACAGCTGCTCCCTGATGAAGATTTCATAAGGAGTTGGTAGTCTCTGGGAAATGGGCTGATATATTAGAGGTCAATTTCAAAAACTGCAACATTTCTCCTAGGAGAATCCAGGCAATGTGTGCACTGCTCTAACCCCACTGAGAACCCTGACATCTCAAAATGAAGGGACTAATTAAAATGGTGGCATTGGTCCTTGCCTGGCCTTTGTTGTGTGATGTTGACACCTTCCTCATAGATCAGAAATCTCACAGAGACACTGCCTCTGTGACAATTGAAGCCAGAGAGCCAAACAGACAATCTTACAAAAGCCATGAGACGCTCTAATCACTAAACTACAGGATACGTGAACTTGGAATTGTGCAAGCATGGCCTCTACTTGAAAGTGGACTATAACAAAAGAAACTTTATTTACCTCAACTTTTCTGAGTTCATGTCCAGGTGTCAATACTTTTCAGCACACCTTAACTAACACAAATATTTGAAATCCAAAATTCTCAGAAAGAAATGTTAAGACGCTTAACTTCAAAAATCAAAAATTGCGGATATTCACTTTTGGCACATAACTTCCCTGAAAACACACACCAAACAAATATTAAACAACACATACAGTGAACACTACATTTAAGAGCAGTTATATTGTTACTGTTTTAAGTCAAAATTCAAGAGCTTAAAAAATGCCCAACATATATATTCCATTGCAATGTATTCTTAGATTGTTTTCAGGCTTGATCAAATAAACATGGAATTTGTGGAAATATATTTTAAAAATCTATTTATCATTTTCTCTCCCAATTCACAATTAACTTGCGACTTATGAGGAACTAAAAAAATAAAATGAATGCCTGGTTATATTCACATTTATTAACTAAATATTATTCCAACTTTAGAGTTAATGTTAAATGGATTTGAACTGTAATGGCTAATATTTGGAAAAATCTATTAAAAGTATTAGCAGTGTGGCTGGGTTCTGTTTCTTGTGTTATATGTCATACTATTAGTATCATACAATTAAGTCTTCAAATGTTTTAAAAATAAACCATATATTTGATGGTGTTTAAG, which encoded the M1 isoform of AQP4.

The sequence of *Runx1* cDNA was: 5’-tctagaATGGCTTCAGACAGCATTTTTGAGTCATTTCCTTCATATCCACAGTGCTTCATGAGAGATGCCAGCACGAGCCGCCGCTTCACGCCGCCTTCCACCGCGCTGAGCCCCGGCAAGATGAGCGAGGCGCTGCCGCTGGGCGCCCCGGATGGCGGCGCCGCCCTGGCCAGCAAGCTGAGGAGCGGCGACCGCAGCATGGTGGAGGTACTAGCTGACCACCCTGGCGAGCTAGTGCGCACCGACAGCCCCAACTTCCTCTGCTCCGTGCTACCCACTCACTGGCGCTGCAACAAGACCCTGCCCATCGCTTTCAAGGTGGTGGCACTGGGGGACGTCCCGGATGGCACTCTGGTCACCGTCATGGCAGGCAACGATGAAAACTACTCGGCAGAACTGAGAAATGCTACCGCGGCCATGAAGAACCAGGTAGCGAGATTCAACGACCTCAGGTTTGTCGGGCGGAGCGGTAGAGGCAAGAGCTTCACTCTGACCATCACCGTCTTTACAAATCCGCCACAAGTTGCCACCTACCATAGAGCCATCAAAATCACAGTGGACGGCCCCCGAGAACCCCGAAGACATCGGCAGAAACTAGATGATCAGACCAAGCCCGGGAGTTTGTCCTTTTCCGAGCGGCTCAGTGAATTGGAGCAGCTGCGGCGCACGGCCATGAGGGTCAGCCCGCACCACCCAGCCCCCACGCCCAACCCTCGGGCCTCCTTGAACCACTCCACTGCCTTTAACCCTCAGCCTCAAAGTCAGATGCAGGATGCCAGGCAGATCCAGCCATCCCCACCGTGGTCCTATGACCAGTCCTACCAGTACCTGGGATCCATCACCTCTTCCTCTGTCCACCCAGCGACACCCATTTCACCCGGCCGTGCCAGCGGCATGACCAGCCTCTCTGCAGAACTTTCCAGTCGACTCTCAACGGCTCCGGACCTGACCGCCTTCGGCGACCCACGCCAGTTCCCTACTCTGCCGTCCATCTCCGACCCGCGCATGCACTACCCAGGCGCCTTCACCTACTCGCCGCCCGTCACGTCGGGCATCGGCATCGGCATGTCAGCCATGAGCTCGGCCTCTCGCTACCACACCTACCTGCCGCCGCCCTACCCCGGCTCATCACAGGCGCAGGCCGGGCCCTTCCAGACCGGCTCGCCCTCCTACCATCTATACTACGGCGCCTCGGCCGGTTCCTACCAGTTCTCCATGGTGGGCGGAGAGAGATCGCCCCCGCGCATCCTGCCGCCCTGCACCAACGCATCCACCGGCGCCGCGCTGCTCAACCCCAGCCTCCCCAGCCAGAGCGACGTGGTGGAGACCGAGGGCAGCCATAGCAACTCGCCCACCAACATGCCCCCCGCGCGCCTGGAGGAGGCCGTGTGGCGGCCCTACTGAgaattc-3’.

The sequence of *Cmyb* cDNA was: 5’-ggatccATGGCCCGGAGACCCCGACACAGCATCTACAGTAGCGATGAAGATGATGAAGACATTGAGATGTGTGACCATGACTACGATGGGCTGCTGCCCAAATCTGGAAAGCGTCACTTGGGGAAAACTAGGTGGACAAGGGAAGAGGATGAGAAGCTGAAGAAGCTGGTGGAACAGAACGGAACAGACGACTGGAAAGTCATTGCCAATTATCTGCCCAACCGGACAGATGTGCAGTGCCAACACCGGTGGCAGAAAGTGCTGAACCCTGAACTCATCAAAGGTCCCTGGACCAAAGAAGAAGATCAGAGAGTCATAGAGCTTGTCCAGAAATATGGTCCGAAGCGTTGGTCTGTTATTGCCAAGCACTTAAAAGGGAGAATTGGAAAGCAGTGTCGGGAGAGGTGGCACAACCATTTGAATCCAGAAGTTAAGAAAACCTCCTGGACAGAAGAGGAGGACAGAATCATTTACCAGGCACACAAGCGTCTGGGGAACAGATGGGCAGAGATCGCAAAGCTGCTGCCCGGACGGACTGATAATGCTATCAAGAACCACTGGAATTCCACCATGCGTCGCAAGGTGGAACAGGAAGGCTACCTGCAGGAGCCTTCCAAAGCCAGCCAGACGCCAGTGGCCACGAGCTTCCAGAAGAACAATCATTTGATGGGGTTTGGGCATGCCTCACCTCCATCTCAGCTCTCTCCAAGTGGCCAGTCCTCCGTCAACAGCGAATATCCCTATTACCACATCGCCGAAGCACAAAACATCTCCAGTCACGTTCCCTATCCTGTCGCATTGCATGTTAATATAGTCAACGTCCCTCAGCCGGCTGCGGCAGCCATCCAGAGACACTATAACGACGAAGACCCTGAGAAGGAAAAGCGAATAAAGGAGCTGGAGTTGCTCCTGATGTCAACAGAGAACGAGCTGAAGGGACAGCAGGCATTACCAACACAGAACCACACTTGCAGCTACCCCGGGTGGCACAGCACCTCCATTGTGGACCAGACCAGACCTCATGGGGATAGTGCACCTGTTTCCTGTTTGGGAGAACACCATGCCACCCCATCTCTGCCTGCAGATCCCGGCTCCCTACCTGAAGAAAGTGCCTCACCAGCAAGGTGCATGATCGTCCACCAGGGCACCATTCTGGACAATGTTAAGAACCTCTTAGAATTTGCAGAAACACTCCAGTTTATAGATTCTGATTCTTCGTGGTGTGATCTCAGCAGTTTTGAATTCTCTGAAGAAGCGGCAGCTTTTTCACCTAGCCAGCAGCCCACAGGCAAAGCCTTCCAGCTTCAGCAAAGAGAGGGCCATGGGACTAGATCTGCAGGAGAGCCTAGCCTGAGGGTGACCAGGCGAGTGCTGAGCGAGGCATCCCTCGGCCCAGACTCACCCCAAGCGAGGCACAGCAAGGTTCCGCTGGTCGTCCTACGAAAAAGGCGGGGCCAGGCCAGCCCCCTAGCCGCTGGAGAGCCTAGCCCCTCCCTCTTTGCTGACGTCATCAGCTCAACTCTCAAGCGTTCCCCTGTCAAAAGCCTACCCTTCTCTCCCTCGCAGTTCTTGAACACTTCCAGCAACCATGAAAGCTCGGGCTTAGATGCACCTACCTTACCCTCCACTCCTCTCATTGGTCACAAACTGACACCATGTCGAGACCAGACTGTGAAAACCCAGAAGGAAAATTCCATCTTTAGAACTCCAGCTATCAAAAGGTCAATCCTCGAAAGCTCTCCTCGAACTCCCACACCATTCAAACATGCCCTTGCAGCTCAAGAAATTAAATACGGTCCCCTGAAGATGCTACCTCAGACCCCCTCCCATGCAGTGGAGGACCTACAAGATGTGATTAAGCAGGAATCGGATGAATCTGGAATTGTGGCTGAGTTTCAAGAGAGTGGACCACCGTTACTGAAAAAAATCAAGCAGGAGGTGGAGTCGCCAACTGAGAAATCGGGAAACTTCTTCTGCTCAAACCACTGGGCAGAGAACAGCCTGAGCACCCAGCTGTTCTCGCAGGCGTCTCCTGTGGCAGATGCCCCAAATATTCTTACAAGCTCTGTTTTAATGACACCTGTATCAGAAGATGAAGACAATGTCCTCAAAGCCTTTACCGTACCTAAGAACAGGCCCCTGGTGGGTCCCTTGCAGCCATGCAGTGGTGCCTGGGAGCCAGCATCCTGTGGGAAGACAGAGGACCAGATGACGGCCTCCGGTCCGGCTCGGAAATACGTGAACGCGTTCTCAGCTCGAACTCTGGTCATGTGAagcggccgc-3’.
